# Human milk banking services: an umbrella review

**DOI:** 10.1017/S1368980026103231

**Published:** 2026-07-27

**Authors:** Roisin O’Donovan, Margaret Murphy, Elaine Lehane, Edina Hanley, Jane Bourke, Liz Cogan, Jennifer Hanratty, Naomi Hurley, Aoife Long, Helen Mulcahy, Susan Rogers, Siobhan Ward, Gillian Weaver, Patricia Leahy-Warren

**Affiliations:** 1 University College Cork, Ireland; 2 The Centre for Effective Services, Ireland; 3 Childhood Development Initiative, Tallaght, Dublin 24, Ireland; 4 HSC Western Health and Social Care Trust, UK; 5 La Leche League, Ireland; 6 The Human Milk Foundation, UK

**Keywords:** Human milk banking, Umbrella review, Donor human milk, Breast milk, Lactation

## Abstract

**Objective:**

Donor human milk (DHM) is excess breast milk a woman donates to a human milk bank (HMB) for use by another infant. HMB requires operational sustainability to ensure consistent and equitable DHM supply. This review synthesises evidence from systematic reviews on how HMB services have been developed and scaled internationally. Results will inform the co-design of an evidence framework that incorporates governance, quality and sustainability criteria for HM banking.

**Design:**

An umbrella review was conducted following the JBI Manual for Evidence Synthesis. CINAHL, MEDLINE, PsycINFO, Embase, JBI Evidence Synthesis, Epistemonikos and PROSPERO datasbases were searched along with grey literature searches using Google Scholar, BASE and the National Institute for Health and Care Excellence website. Studies published between 2000 and June 2025 were screened by two independent reviewers.

**Setting:**

HMB or HMB service operating in any middle- and high-income country.

**Participants:**

Prospective, current or past HM donors; donor milk recipient families; staff working within HMB service.

**Results:**

Thirteen reviews were identified. Limited information on HMB organisational and funding models were identified. Key stakeholders include DHM donors and recipients, healthcare professionals and social supports. Institutional support and providing information about HMB were found to be facilitators of DHM donation and acceptance of DHM among recipient parents. Barriers to HMB development included limited public information, religious beliefs and lack of support.

**Conclusions:**

A significant information gap was identified in HMB funding and organisational models. Establishing sustainable funding models, standardised governance structures and strategies to improve healthcare professionals knowledge and public awareness are opportunities for future research.

The WHO recommends that donor human milk (DHM), obtained from a human milk bank (HMB) facility, be available to all preterm and low birthweight infants when there is insufficient milk available from their mother^(1,2)^. DHM is human milk (HM) in excess of an infant’s current and future needs that is donated by an infant’s mother^(3)^. When supplementation is required, DHM is the preferred alternative to mother’s own milk (MOM). DHM should serve as a bridge to ensure an exclusively HM diet by providing lactation support to build a mother’s milk supply^(3)^ or, where needed, as an infant’s sole source of HM.

HM is recognised as a pillar of child survival^(4)^ and is the optimal diet for preterm infants^(5)^. It delivers a wide range of nutritional, immunological, neurological and physiological benefits to infants^(6,7)^. An exclusively HM diet during the first days of life is associated with decreased infection, morbidity and mortality in very low birth weight infants^(8–12)^. Preterm infants who are exclusively fed HM are less likely to experience necrotising enterocolitis compared with formula fed infants^(9,10,13)^. However, preterm and low birth weight infants often lack sufficient access to their mother’s milk during the first hours, days, weeks or even months of life^(6,14)^. Women who deliver babies prematurely can encounter a variety of complications that impact their ability to produce a full supply of milk. They may be severely ill or recovering from emergency interventions at birth. They are often separated from their infants at birth and must learn how to establish and maintain milk supply through expression. Provision of DHM to preterm or low birthweight infants supports an exclusive HM diet while maternal lactation is being established. In this way, DHM acts as a bridge to establish continued and future breast-feeding. This requires support both in the hospital and after they are discharged to establish and sustain exclusive breast-feeding^(6,14)^.

There are an estimated 800 HMB operating globally in approximately seventy countries^(15)^. HMB require operational sustainability to ensure consistent and equitable of DHM supply. Sustainable operation of HMB requires a stable balance of DHM supply and demand, financial viability and robust communication, policy and advocacy frameworks^(6)^. Strategies to enhance sustainability include supporting lactating mothers to maintain a consistent donor base, fostering local stakeholder engagement and ensuring effective financial and non-profit governance^(6,16)^. Community education and engagement can raise public awareness of HMB activities and normalise HM feeding for all infants^(6)^.

Ensuring sustainable service provision requires equitable recruitment of donors and provision of DHM. Lack of equal opportunity to donate milk and access DHM exist globally^(6,17)^. Providing payments or reimbursements to milk providers is atypical and is illegal in some countries, however, informal peer to peer channels (i.e. the internet) mean that it is often tolerated outside of HMB^(17,18)^. The absence of financial or other incentives minimises the chance of donors deliberately concealing information (e.g. drug, tobacco and alcohol use) that may impact the quality and safety of their milk. Providing adequate support and accurate information to enable informed consent is vital to avoid exploitation of HM donors and their infants^(17)^. Within HMB, DHM is typically allocated based on the risk of morbidity and mortality, prioritising the smallest and sickest infants to avoid the potential health risks associated with formula use^(6)^.

Integration of HMB services with comprehensive breast-feeding support is essential to optimise infant nutrition and health outcomes. Within the broader healthcare system, this involves provision of professional lactation support, minimising mother–infant separation and adhering to the UNICEF Baby Friendly Initiative standards for breast-feeding promotion^(17)^. An enabling social, cultural and policy environment is essential to protect and promote breast-feeding at a population level^(19)^. Establishing such supportive conditions for maternal lactation and breast-feeding ensures the sustainability of DHM provision^(6)^.

Free online resources, including a practical toolkit, are available to help establish and integrate HMB with breast-feeding support and neonatal care^(20)^. These resources provide in-depth guidance on readiness, quality assurance, operations, auditing, training, evaluation and communications. In May 2024, the EU Council adopted new rules aimed at improving the safety and quality of substances of human origin. In alignment with this, the EU-funded project (IMAGINE-HMB Implementation of HM hArmonized Guidelines for Infant Nutrition in Europe) is developing evidence-based guidelines to support the safe and standardised provision of DHM across Europe. This initiative, grounded in multidisciplinary collaboration, builds on recent reviews addressing regulatory evidence-based practice needs^(21–23)^.

This umbrella review will synthesise evidence from systematic reviews on how HMB services have been developed and scaled to inform the co-design of an evidence framework that incorporates governance, quality and sustainability criteria for a proposed milk banking resource framework.

## Methods

### Research questions

The overarching research question: What is the current state of the art in the development and national-level implementation of HMB service, including operational models, implementation and integration into healthcare systems?

The specific research questions:

- How are HMB defined?
- How are HMB networks funded, developed and scaled at a national levels?
- What organisational models are used in the implementation of milk banks?
- What distribution/access models are used for DHM and how are they impacted by DHM supply, available guidelines and decision-making of clinical staff?
- What roles do key stakeholders play in supporting and sustaining milk bank networks?
- What are the facilitators and barriers to the implementation of milk banks in middle- and high-income countries?

### Protocol and registration

A review protocol was developed and registered on Prospero. The review protocol was published on Health Research Board Open Research (references removed for anonymity).

### Design

This review followed the guidelines for umbrella reviews from the JBI Manual for Evidence Synthesis^(24)^. An umbrella review is a systematic review of existing systematic reviews conducted to compare these existing reviews^(24)^. This enables synthesis of the highest level of existing evidence to inform the co-design of an evidence framework for a national milk banking resource framework for Ireland.

### Inclusion criteria

Systematic reviews of studies reporting on the development and implementation of a milk banking service. Eligibility criteria are outlined in Table 1. Reviews published in English since 2000 were considered for inclusion.

**Table 1.** Eligibility criteria Table 1 long description.

Inclusion criteria
**Participants** Prospective, current or past human milk donors; donor milk recipient families; staff working within HMB service. **Phenomena of interest** This review focused on HMB service and the provision of DHM. **Outcomes** The acceptability, development, implementation and maintenance of affordable, effective, efficient, safe and sustainable HMB service. **Context/Setting** HMB or HMB service operating in any middle- and high-income country. **Types of studies** Existing systematic reviews and meta-analyses. All types of syntheses of research evidence were included, including systematic reviews in their various forms (effectiveness, meta-aggregative, integrative, meta-synthesis, etc.) and meta-analyses.
**Exclusion criteria**
Reviews that did not report on the development or implementation of a HMB service. Reviews not available in English.

### Search strategy

A three-phase search process was used to locate both published and unpublished reviews^(24)^. An initial search was carried out on PubMed, CINAHL and Medline to identify articles on the topic. The initial keywords were informed by the Special Issue of Maternal and Child Nutrition Volume 20: Expert overview of critical focus areas towards the development of global HMB standards from July 2024.

The keywords contained in the Special Issue were used to develop a full search strategy (Tables 2 and 3). The search strategy was reviewed by topic experts and knowledge users who are professionals in this area.

**Table 2.** Developing the search strategy Table 2 long description.

Search concepts	Human milk bank service	Synthesis studies
Search terms	‘donor milk’ OR ‘milk bank*’ OR ‘milkbank*’ OR ‘human milk bank*’ OR ‘donor human milk’ OR ‘milk donation’ OR ‘donor breastmilk’ OR ‘donor breast milk’ OR ‘human donor milk’ OR ‘human milk donor’ OR ‘Breast* milk bank*’ OR ‘human milk shar*’ OR ‘milk shar*’ OR ‘milk kinship’ OR ‘nurs* milk’ OR ‘cross* milk’ OR ‘milk sibling*’	‘systematic’ OR ‘meta- analysis’ OR ‘meta-aggregative’ ‘integrative review’ OR ‘meta-synthesis’

**Table 3.** Search strategy Table 3 long description.

Search	Query
#1	**Title/Abstract:** ‘donor milk’ OR ‘milk bank*’ OR ‘milkbank*’ OR ‘human milk bank*’ OR ‘donor human milk’ OR ‘milk donation’ OR ‘donor breastmilk’ OR ‘donor breast milk’ OR ‘human donor milk’ OR ‘human milk donor’ OR ‘Breast* milk bank*’ OR ‘human milk shar*’ OR ‘milk shar*’ OR ‘milk kinship’ OR ‘nurs* milk’ OR ‘cross* milk’ OR ‘milk sibling*’
#2	**Title/Abstract:** ‘systematic’ OR ‘meta- analysis’ OR ‘meta-aggregative’ ‘integrative review’ OR ‘meta-synthesis’
#3	#1 AND #2
Limits	2000-present

Database-specific searches were conducted for CINAHL (EBSCO), MEDLINE (EBSCO), PsycINFO (EBSCO), PubMed and Embase (Ovid). JBI Evidence Synthesis, Epistemonikos and the PROSPERO register were searched. A grey literature search was undertaken on Google scholar, BASE and the National Institute for Health and Care Excellence website. All database and grey literature searches were conducted on 03 June 2025. The reference lists of all included reviews were searched on 08 August 2025. Date restrictions (2000-current) were used. The full search has been included (Table 4).

**Table 4.** Full sample search string for CINAHL Table 4 long description.

Search	Query	Records retrieved
#1	**Title/Abstract:** ‘donor milk’ OR ‘milk bank*’ OR ‘milkbank*’ OR ‘human milk bank*’ OR ‘donor human milk’ OR ‘milk donation’ OR ‘donor breastmilk’ OR ‘donor breast milk’ OR ‘human donor milk’ OR ‘human milk donor’ OR ‘Breast* milk bank*’ OR ‘human milk shar*’ OR ‘milk shar*’ OR ‘milk kinship’ OR ‘nurs* milk’ OR ‘cross* milk’ OR ‘milk sibling*’	1222
#2	**Title/Abstract:** ‘systematic’ OR ‘meta- analysis’ OR ‘meta-aggregative’ ‘integrative review’ OR ‘meta-synthesis’	269 543
#3	#1 AND #2	48
Limits	2000-present	

### Screening

**Figure 1.**
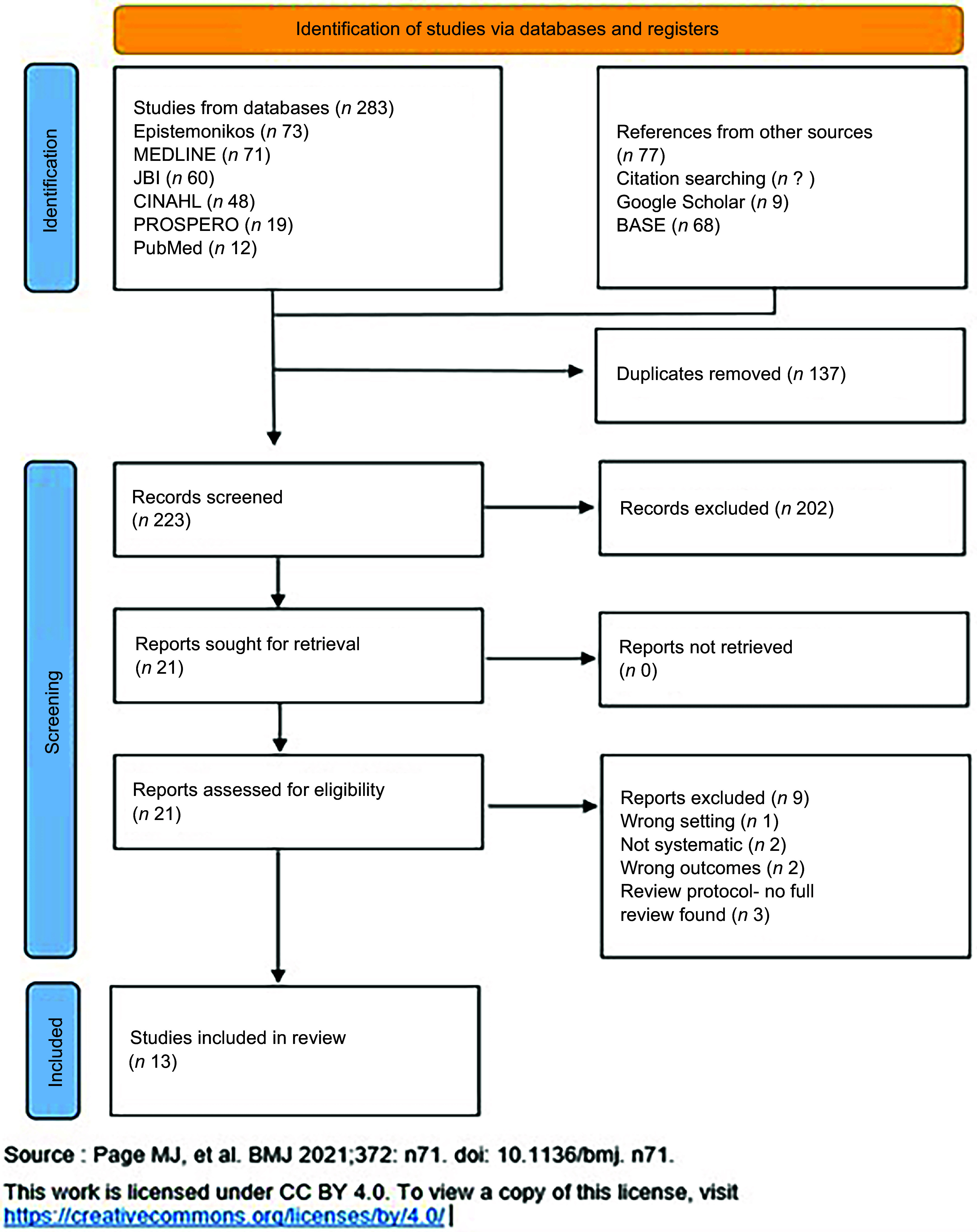
Figure 1 long description. PRISMA flow diagram.

Records identified through database searching (*n* 283) and grey literature search (*n* 77) were exported into Covidence and duplicates removed (*n* 137). Title, abstract and full texts were screened using Covidence, a web-based systematic review software platform. Two reviewers (ROD and EH) independently screened the titles and abstracts of 223 records for assessment against the eligibility criteria (Table 1) with 202 records excluded. A third reviewer (GW) was consulted where disagreement arose, and consensus was reached through discussion. The full text of the remaining twenty-one articles was assessed independently by two reviewers (ROD and EH), and thirteen articles met the inclusion criteria. Where protocols were identified, authors were contacted to identify the status of the review, one author returned the full text of their recently published article and the other three were ongoing. Reasons for article exclusion are reported in Figure 1. Results of the screening process are presented in a PRISMA flow diagram in Figure 1 ^(25)^.

### Data extraction

Two reviewers independently conducted data extraction (ROD, EH) using a piloted and revised data extraction template based on the standardised JBI data extraction tools, adapted to align with the research questions (see Table 5). Disagreements between reviewers were resolved through discussion (ROD, EH), and a third reviewer was not required.

**Table 5.** Data extraction instrument Table 5 long description.

Author/year	Objectives	Participants	Setting/Context	Search details	Role of key stakeholders	Development/implementation process, facilitators, barriers	Funding source/type	Factors impacting Sustainability	Donor experience

### Data analysis

All extracted data were mapped according to the aims and objectives of this review.

We adopted a reflexive approach throughout the review process. Data analysis involved reviewers engaging in critical reflection throughout all the stages. Reviewers were conscious of the potential biases inherent in the synthesis process.

### Quality assessment

The JBI critical appraisal instrument for Systematic reviews and Research Syntheses was used by two reviewers independently (ROD and EH) to assess the quality of reviews.

## Results

**Figure 2.**
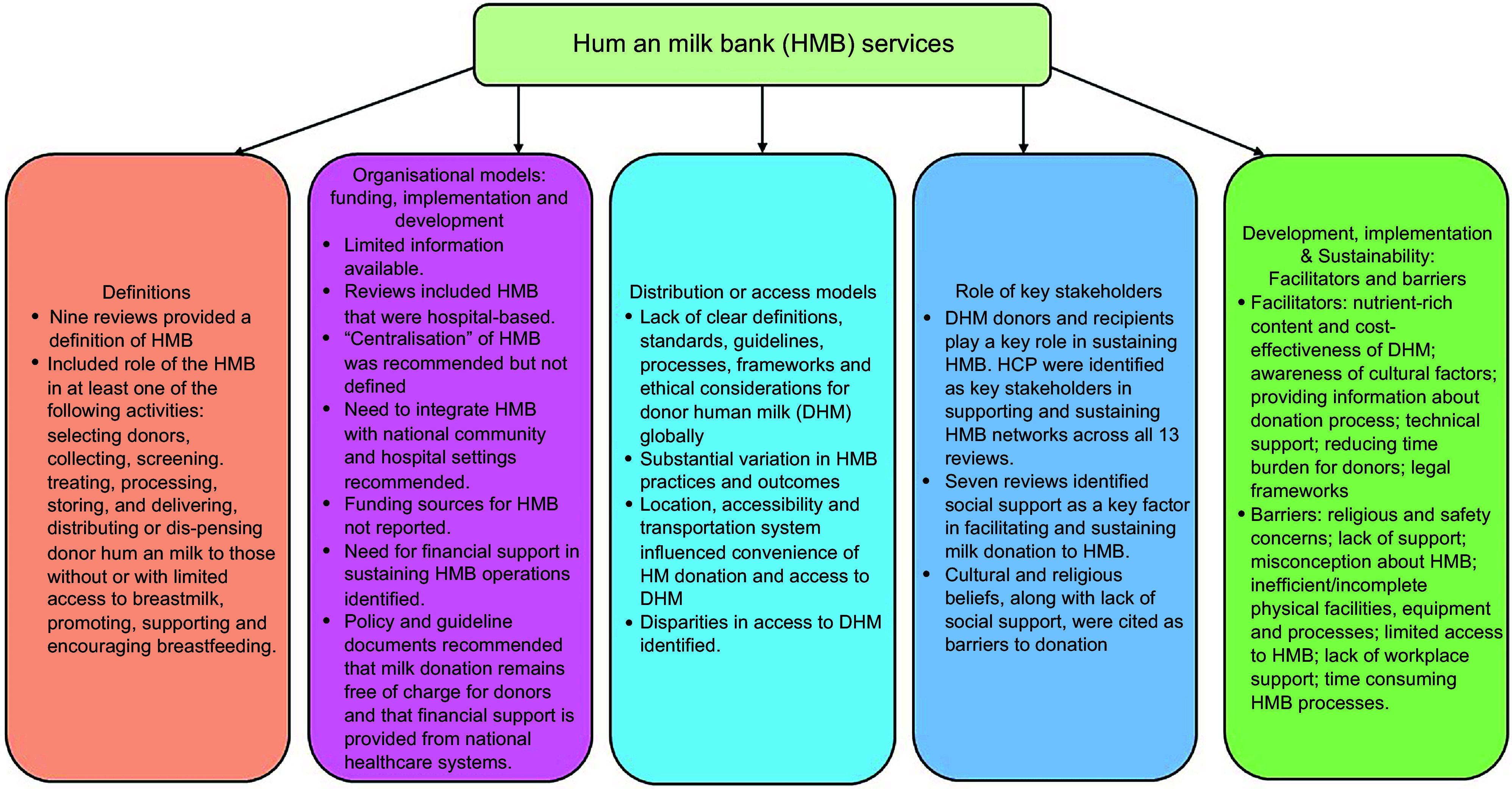
Summary of results.

A visual summary of our results can be found in Figure 2.

### Description of included reviews

Of the thirteen reviews that met inclusion criteria, seven were systematic reviews^(21–23,26–29)^, three were scoping reviews^(30–32)^, two were integrative reviews^(33,34)^ and one was a systematic document analysis^(35)^. Included reviews were published between 2014 and 2025 and included between seven^(21,34)^ and sixty-three^(26)^ primary studies. Of the eleven reviews that clearly reported individual studies, twenty-nine studies were included in more than one review. A total of 108 individual studies and eight policy or guideline documents relating to the sustainability of HMB were included in a single review. The individual studies included in two reviews (Rechi *et al.* ^(33)^ (*n* 20); Badrul Hisham *et al.* ^(26)^ (*n* 63)) were not clearly reported, making it impossible to determine whether there was overlap with other reviews. The total number of participants was not clearly reported in five reviews^(22,26,27,31,33)^ and was not applicable to one review that included only policy and guidelines documents^(35)^. Among reviews that did report participants numbers, sample sizes ranged from 1014^(28)^ to 7053^(23)^ and included donors, non-donors and healthcare professionals (HCP).

The included reviews encompassed studies conducted across both high- and low-to-middle-income countries. Four reviews contained studies exclusively from middle- and high-income countries^(21,27,32,34)^, while two did not clearly report study locations^(26,33)^. Five reviews included studies from countries with varying income levels, each containing at least one from a lower-middle income country^(22,23,28,29,31)^. One review focused on global and European policy and guideline documents^(35)^. As the aim of this umbrella review was to synthesise the findings of systematic reviews rather than individual studies, no exclusions were made based on country income level. Consequently, the results are not limited to middle- and high-income countries, as initially specified in our study protocol. This change does not significantly affect the review’s objectives because they are focused on issues of universal relevance and importance, such as governance, funding and sustainability of HMB.

Details of included reviews are reported in Table 6

**Table 6.** Review details Table 6 long description.

Author/Year/Review type	Objectives	Participants (characteristics/total number)	Search details (sources searched/inclusion criteria/date range/)	Included studies: number/design/country
Badrul Hisham *et al.*, 2024 Systematic Review	To unravel the complexities surrounding the policies governing breast MB. By scrutinising regulatory landscapes, socio-economic considerations, healthcare integration and technological interfaces, the review aims to provide a comprehensive foundation for future policy recommendations.	Not clearly reported	Scopus and Web of Science/One primary criterion involved prioritising literature, particularly research articles, as the primary source of practical information/published in English/2019–2023.	*n* 63/Not clearly reported/ Not clearly reported.
Doshmangir *et al.* 2019 Systematic Review	To systematically review the factors that affect donations to HMB.	Total (*n* 6057) According to Table 1: Donors (*n* 1143) Potential donors (*n* 5) Recipients (*n* 41) Women enrolled in two HMB (*n* 72) Mothers who are BF, pregnant or have a child under 1 (*n* 3027) Mothers who gave birth (*n* 1686) Mothers (*n* 24) Milk sharers (*n* 981) Non donors (*n* 19) Women (*n* 453) Married women (*n* 231) Grandmothers (*n* 5) Partners (*n* 4) Healthcare professionals (*n* 19) Population not stated (*n* 948) Total according to table (*n* 8658) Donor characteristics: Mean age of participants’ range: 24.73–42 years. Majority were educated and had 1–3 children.	PubMed, Scopus, Embase, ScienceDirect, and Web of Science on 16 December 2018. Reference lists of included studies and google scholar were searched/English language, peer reviewed published studies were included with no time limitation. Publications of any level of evidence, including abstracts, conference proceedings, commentaries and editorial articles, and full text of original articles and systematic reviews were included in the review. Other studies that did not focus on the factors related to the donated milk, donors and the reasons for donation were excluded.	*n* 31/Cross-sectional (*n* 12), Quantitative (*n* 1), Qualitative (*n* 6), Descriptive (*n* 3), Case report (*n* 2), Review (*n* 5), Retrospective (*n* 1), Mixed-methods (*n* 1)/Mixed income countries: United States (*n* 5), Brazil (*n* 5), Australia (*n* 3), France (*n* 3), Kuwait (*n* 2), Spain (*n* 2), Turkey (*n* 5), South Africa, India, Ethiopia, Nigeria, Canada, UK (all *n* 1)
Dos Santos & Perrin 2021 Scoping Review	To explore what is currently known about HMB donors globally and identify gaps for future research.	Donors (*n* 8522) Donors to sixteen MBs from 2015 to 2016 (range 70–4000 per bank) Potential/prospective donors (*n* 4604) Non-Donors (*n* 663) Recipients (*n* 19) Donors’ online testimonial (*n* 95) and images (*n* 107) Donor characteristics: variation with donor being both primiparous and multiparous and including those who had preterm and term births; limited research regarding donors’ lifestyle characteristics including diet, exercise, legal and illegal drug use. Beliefs about the importance of breast-feeding and breast milk across three geographies (*n* 3); Donors expressed desire for compensation. Limited information about donors’ BF history, clinical support for lactation and milk expression practices.	PubMed and Scopus, hand searched reference lists of identified studies/Original research articles about MB donors that were published before August 2020 were included in this review. Studies were excluded if they were (1) about DM composition and/or pasteurisation only, (2) about infant feeding practices and/or infant nutrition only, (3) in languages that were not English, (4) not original articles or (5) not about MB donors (e.g. peer-milk sharing only).	28/Cross-sectional (*n* 7), Qualitative (*n* 8), Case–control (*n* 2), Semi-longitudinal (*n* 10), Not identified (*n* 1)/Mixed income countries: USA (*n* 8), Brazil (*n* 7), India (*n* 2), Spain (*n* 4), France, Norway, Poland, Italy, Taiwan, Korea and China (all *n* 1)
Fonseca *et al.*, 2021 Systematic Review	With the aim of supporting and drawing together theoretical aspects of the activities developed by HMB that demonstrate the role these facilities play in promoting maternal and infant health.	Not reported.	PubMed, Virtual Health Library (BVS) and BVS BF, linked to the portal of the RHMB. References of identified articles searched/full-text scientific articles in English, Spanish and/or Portuguese that addressed the guiding question or contained data on the development (or absence) of educational and BF promotion actions. Duplicate articles, theses, dissertations and editorials were excluded. No date restriction.	11/Cross sectional (*n* 6), qualitative (*n* 3), quantitative descriptive, comparative, prospective (*n* 1), retrospective study (*n* 1)/ middle- and high-income countries: Brazil (*n* 11)
Kaech *et al.*, 2022 Systematic Review	To assess and synthesise contemporary evidence on the factors that sustain the donation of HM to not-for-profit HMB by existing lactating donors. The factors influencing donation sustainability are explored at the micro (individual, donor), meso (institutional) and macro (systemic) levels and include donor characteristics and behaviours, professional practices, organisational structures and donation practices, guidelines, regulations and policy.	Donors (*n* 6879) Non-donors (*n* 19) Service providers (*n* 9) Service recipients, including HM donors and key influencers (*n* 56) Enrolled donors (*n* 90) Total (*n* 7053)	MEDLINE, CINAHL, EMBASE, Cochrane Library, CENTRAL, SCOPUS, PsycINFO and Web of Science/ the inclusion criteria were primary research studies written in English or French that investigated factors influencing the sustainability of HM donation to HMB. The study population comprised HM donors or any stakeholders involved in HMB. The exclusion criteria were studies (1) for which the methods used were unclear; (2) with no full article available; (3) that focused exclusively on milk sharing for-profit MB or HM sales or (4) that were published before 1990, as the phenomena of interest for those studies were not relevant to this review- range to 2021.	*n* 10/Cross sectional (*n* 7), cohort study (*n* 1), qualitative (*n* 2)/ mixed-income countries: Brazil, India, Italy, Spain and the USA (all *n* 2).
Kaech *et al.* 2025 Qualitative Systematic Document Analysis	Investigate how global (universal), European and national policies and guidelines support the sustainability of human milk donation to human milk banks in Switzerland at the macro, meso and micro levels.	NA-all policies and guideline documents.	Medline (PubMed) and Cumulative Index to Nursing and Allied Health Literature (CINAHL) databases. Manual searches were performed on Google, Google Scholar and the websites of various professional associations, organisations and other entities such as the European Foundation for the Care of Newborn Infants (EFCNI), the European Milk Banking Association (EMBA), the European Directorate for the Quality of Medicines & Healthcare (EDQM) and PATH, among others. The lists of references in the guidelines found during the literature search were also searched to identify potential additional guidelines (snowballing references)/ Current global, European or Swiss national policies and guidelines or recommendations that were focused on supporting (explicitly or implicitly) the sustainability of human milk donation. Only policies and guidelines (including documents self-defined as position papers and toolkits) relevant to Switzerland – meaning they either target users in Switzerland or pertain to the country geographically (e.g. national, European or global) – were included/ No date limits	8/2 guidelines; 1 framework; 1 toolkit; 2 position papers/consensus statements; 1 policy recommendations document; 1 guideline review/ 2 global; 5 European; 1 Swiss
Kundisova *et al.*, 2019 Systematic Review	To undertake a systematic review of literature, regarding the characteristics and motivations of milk donors and to identify factors influencing their willingness to donate their breastmilk. Other variables influencing the willingness to donate were investigated, to introduce measures that would lead to an increase in breastmilk donation.	Donors (*n* 2142); non donors (*n* 119). Total (*n* 2261) Characteristics of Donors: Differences in typical donor profiles were observed between European countries and USA and Brazil. The average age of Brazilian donors was lower in comparison with USA and European countries: in Brazil 22 years; in Italy 30.8, in Spain 30.7 and France 28.5. Higher rates of donors with medium-high education level in USA and European countries in comparison to Brazil. Mixed evidence found on whether higher education impacted likelihood of donation. The highest rates of donors not working outside their homes were reported in Brazil (*n* 3); lowest rates were observed in the USA and Italy (*n* 2). Work status did not influence the likelihood of donation (*n* 1). The differences observed across the studies can be attributed to differences in maternity leave and policies in countries, i.e. donors from the USA are disadvantaged because they do not receive any financial support. Donors were majority multiparous (*n* 2, US and Brazil) or primiparous (*n* 2, Italy and Spain). Younger maternal age (*n* 1) and having a preterm infant (*n* 1) was associated with higher amounts of milk. Admission of the newborn to the neonatal unit was associated with a lower prevalence of donation (*n* 2). 25 % of donors came from health or social fields of work (*n* 1).	PubMed/ Papers that contained the following variables: socio-demographic characteristics of milk donors (country of origin, age, marital status, education level, work status), gynaecologic anamnesis related characteristics (number of pregnancies (parity), type of labour (preterm/full-term)) and information related to donation (reasons and motivations to donate, length of donation period, the average volume of donated milk), the information sources that provided milk donors with adequate information about the possibility of donation were examined.	*n* 7/Cross-sectional (*n* 7)/ Middle- high income countries: Brazil (*n* 3), France, Italy, Spain, USA (all *n* 1).
Li *et al.*, 2024 Systematic Review	To examine and synthesise the views and experiences of women, donors, recipient mothers and healthcare professionals regarding HM donation or sharing.	Donors (*n* 108) Recipients (*n* 75) Informal milk sharers (*n* 41) Informal milk recipients/used informal milk sharing (*n* 23) Mothers seeking DHM (*n* 1) Women (*n* 99) Service providers and HCP (*n* 150) HMB leaders (*n* 80) Mothers (*n* 296) Mothers and healthcare professionals (*n* 42) Mothers who were permanently or temporarily deferred from donating (*n* 10) Partners or fathers (*n* 5) Grandmothers (*n* 28) Service recipients including mothers and key influencers (*n* 56) Total (*n* 1014)	MEDLINE, CINAHL, Embase, PsycINFO, Web of Science and Scopus and reference lists of selected studies/Included studies that focus on the perceptions and experiences of primiparous and multiparous women, HM donors, recipient mothers and healthcare professionals regarding HM donation or sharing. Included relevant studies published in English and in peer reviewed journals from the inception of the database until 2024. Qualitative primary research studies (including but not limited to designs such as grounded theory, phenomenology, ethnography and action research) were included. Excluded quantitative studies and mixed-methods studies.	*n* 41/Qualitative (*n* 21); phenomenological study (*n* 8) transversal study (*n* 1), grounded theory (*n* 2), descriptive *n* 5), case study (*n* 4)/mixed income countries: United States (*n* 17), Canada (*n* 4), Australia (*n* 5), New Zealand, Brazil, Spain, India, South Africa, Uganda (all *n* 2), United Kingdom, Ireland, Africa, Vietnam, Singapore, Turkey, Malaysia, Chile, (all *n* 1).
Mathias *et al.*, 2023 Scoping Review	To identify the barriers and facilitators for breast milk donation and acceptance among mothers globally.	Donors (*n* 220) Recipients (*n* 148) Milk sharing recipient (*n* 267) Mothers (*n* 795) HCP or service providers (*n* 57) Caregivers (including grandmothers) (*n* 45) Service recipients including mothers and key influencers (*n* 56) Women (*n* 28) No sample size reported in 3 studies. Total (*n* 1616)	PubMed (NCBI), CINAHL (EBSCO), Web of Science (Clarivate) and Embase (Elsevier) were searched on 07/12/2022/. Included studies conducted on mothers of all age groups and ethnicities. Studies conducted on families/caregivers who cared for a BF child or infant with a history of maternal mortality were considered. Studies that restricted mothers from BF their children to prevent transmission of infectious pathogens were considered. Included studies conducted globally (low-, middle- and high-income countries) in hospitals, homes, daycare centres and nursing homes, irrespective of geographical boundaries. included studies reporting barriers and facilitators for breast milk donation and acceptance. Quantitative studies, irrespective of designs, were included. Protocols, editorials, social media posts and magazine reports were excluded as they do not offer substantial information to synthesise. We excluded records where full texts were not available. Only English language literature was included. Between 2000 and 2022.	20/mixed methods studies (*n* 7), descriptive cross-sectional surveys (*n* 7), qualitative studies (*n* 4), not reported (*n* 2)/ mixed income countries: USA (*n* 7), Turkey (*n* 3), Canada (*n* 2), Sweden, Kenya, Uganda, Indonesia, UK, Malasia, Australia, New Zealand, North America, Europe, Oceania and Asia (all *n* 1)
McCune *et al.*, 2021 Scoping Review	Conduct a scoping review of the literature regarding DHM use in populations other than preterm infants.	Adult cancer patients (*n* 5) and family proxies (*n* 5); children fed DHM (*n* 879), children fed formula (*n* 644), children exclusively breastfed (*n* 30); infants with medical needs (*n* 608); foster infants (*n* 8); HIV unexposed infants by feeding type (*n* 94); term NICU infants (*n* 18); late-preterm infants (*n* 28); HCP (*n* 20); mothers (*n* 137); Massachusetts Hospitals and/or hospitals using DHM (*n* 86); preterm quintuplets fed DHM as a supplement to mothers milk at 6 months (*n* 5). Total (*n* 2567)	PubMed and Clinicaltrials.gov/Clinical reports, original research articles, conference abstracts and registered clinical trials. Included: provided DHM to non-preterm infant populations. Excluded: Intended DHM recipients was only premature infants less than 35 weeks gestational age, did not use DHM, was milk sharing only, review article or commentary, not written in English or did not discuss intended recipients (milk only studies). Jan 2000 and Feb2020	26/Observational cohort (*n* 2), observational qualitative (*n* 3), case study (*n* 4), case control, cross sectional observational (*n* 2), Retrospective observational (*n* 5), Observational quality initiative (*n* 1), Observational (*n* 1), Retrospective cohort (*n* 2). Randomised controlled trial (*n* 3)/ Middle- high income countries: USA (*n* 20), Denmark (*n* 2), South Africa, Poland, New Zealand, Canada (all *n* 1)
Nor Azman *et al.*, 2024 Systematic Review	To gather and evaluate evidence on Muslim women’s attitudes, knowledge, perception and views towards breast milk donation and MBs.	Muslim women (*n* 5137) Characteristic of donor: Age between 18 and 45 years old.	Wiley Online Library, Scopus, ScienceDirect, ProQuest and EBSCOHost/Inclusion criteria were primary studies with randomised controlled trial (RCT), non-RCT, observational cohort, case–control, cross-sectional and qualitative study designs that reported on Muslim women’s knowledge, perceptions and views towards breast milk donation and MBs. Exclusion criteria were review articles, conference papers and dissertations, non-English articles and studies that are published as only abstracts without full texts/published between 2016 and November 2023.	12/Quantitative (*n* 11); mixed methods (*n* 1)/ mixed-income countries: Turkey (*n* 10); Syria (*n* 1); Iran (*n* 1).
Rechia *et al.*, 2016 Integrative Review	To analyse the factors that interfere with HM donation in the Brazilian scientific literature.	Mothers/nursing mothers (70 %): Professionals or managers (20 %); Mothers/nursing mothers and professionals (10 %). Total (*n* not reported)	Literatura Latino-Americana em Ciências da Saúde (LILACS), National Library of Medicine (PubMed) and Sci Verse Scopus Top Cited (Scopus)/Original articles in English, Portuguese or Spanish whose theme answered the guiding question: What are the factors that interfere with HM donation, according to Brazilian scientific literature?/No time frame was established/ Search conducted January 2016.	20/Qualitative (*n* 14); quantitative (*n* 5); mixed methods (*n* 1)/not clearly reported.
Sabater *et al.* 2014 Integrative Review	To understand the motivations that lead women to donate milk-to-MBs.	Donors (*n* 1745); non-donors (*n* 19). Total (*n* 1764) Characteristics of donors: Most married, young, economically stable, and healthy; variability in the age of the donors as well as the socio-economic status and educational level. High number of donors do not work outside home compared with the national statistics.	PubMed, CINAHL and VHL (all but 1 included study identified in PubMed)/ Studies of social and motivational aspects of mothers that donated milk to MBs excluding the others/No date range stated in search but stated in results: 1995–2010.	*n* 7/Descriptive study (*n* 2) cross-sectional questionnaire (*n* 2) case–control study (*n* 1) qualitative study (*n* 2)/ middle- high-income countries: Nigeria; France; USA (all *n* 1); Brazil (*n* 4)

### Quality assessment

Overall, quality of included reviews was mixed. Three met all eleven criteria^(23,28,35)^. Two met 9/11^(30)^, one met 8/11^(22)^ two met 7/11^(31,33)^ three met 6/11^(21,27,32)^ and one review met 3/11 criteria^(34)^. One review only met one quality assessment criterion^(26)^. Quality appraisal results are presented in Table 7.

**Table 7.** Quality appraisal Table 7 long description.

Review	Review question stated	Inclusion criteria appropriate to review question	Search strategy appropriate	Adequate sources and resources used to search for studies	Criteria for appraising studies appropriate	Critical appraisal conducted by 2+ independent reviewers	Methods to minimise error in data extraction	Appropriate methods used to combine studies	Likelihood of publication bias assessed	Recommendations for policy and practice supported by data	Specific directives for new research appropriate?
Badrul Hisham *et al.*, 2024	No	Yes	Unclear	No	No	No	Can’t tell	No	No	Can’t tell	Can’t tell
Doshmangir *et al.*, 2019	No	Yes	Yes	Yes	Yes	Yes	Can’t tell	Yes	Yes	Yes	Not reported
Dos Santos & Perrin, 2022	No	Yes	Yes	No	Yes	Yes	Yes	Yes	Yes	Yes	Yes
Fonseca *et al.*, 2021	Yes	Yes	Yes	Yes	No	No	Can’t tell	Can’t tell	No	Yes	Yes
Kaech *et al.*, 2022	Yes	Yes	Yes	Yes	Yes	Yes	Yes	Yes	Yes	Yes	Yes
Kaech *et al.* 2025	Yes	Yes	Yes	Yes	Yes	Yes	Yes	Yes	Yes	Yes	Yes
Kundisova *et al.*, 2019	Yes	Yes	Yes	No	Yes	Can’t tell	Can’t tell	Can’t tell	Yes	Yes	Not reported
Li *et al.*, 2024	Yes	Yes	Yes	Yes	Yes	Yes	Yes	Yes	Yes	Yes	Yes
Mathias *et al.*, 2023	Yes	Yes	Yes	Yes	Not reported	Not reported	Yes	Yes	Not reported	Yes	Not reported
McCune *et al.*, 2021	No	Yes	Yes	No	Can’t tell	Yes	Can’t tell	Can’t tell	Yes	Yes	Yes
Nor Azman *et al.*, 2024	Yes	Yes	Yes	Yes	Yes	Yes	Can’t tell	Yes	Yes	Yes	Not reported
Rechia *et al.* 2016	Yes	Yes	Yes	Yes	Not reported	Not reported	Not reported	Yes	Not reported	Yes	Yes
Sabater *et al.*, 2014	Yes	Yes	Yes	No	Not reported	Not reported	No	Can’t tell	No	Not reported	Not reported

### Definition of human milk bank

Nine reviews provided a definition of HMB (see Table 8). These definitions all refer to the role of the HMB in at least one of the following activities: selecting donors, collecting, screening, treating, processing, storing, and delivering, distributing or dispensing DHM to those without or with limited access to breastmilk. One review defined HMB as being non-profit and providing DHM to preterm infants specifically^(22)^. Five reviews referred to the role of HMB in promoting, supporting and encouraging breast-feeding^(21,22,27,33,35)^. Only one review included the role of HMB in collecting and processing colostrum (first milk produced during pregnancy and first few postnatal days), transitional (breastmilk produced by a breast-feeding mother between the end of colostrum and the beginning of mature milk) and mature milk (breastmilk produced 10–15 d post birth)^(27)^.

**Table 8.** HMB definitions Table 8 long description.

Study	Findings
Badrul Hisham *et al.*, 2024	Not provided.
Doshmangir *et al.*, 2019	Nonprofit health institutions organised for the collection, processing and distribution of donated HM and supporting BF in preterm infants.
Dos Santos & Perrin, 2022	Globally, DHM is typically produced by country-level HMB networks that serve as a conduit between the recipient infants and the donors who provide the milk.
Fonseca *et al.*, 2021	HMB provide support for women having trouble BF and collect, process and control the quality of colostrum, transitional milk and mature milk.
Kaech *et al.*, 2022	HMB are responsible for providing safe DHM. They select and qualify donors and collect, treat (generally by pasteurisation), store and deliver DHM.
Kaech *et al.* 2025	Human milk banks are responsible for collecting, processing and delivering safe DHM to infants in need, while contributing to protecting, promoting and supporting breast-feeding.
Kundisova *et al.*, 2019	Institutions providing human milk (HM) to babies unable to breastfed or with limited access to breastmilk (BM) for various reasons. The HMB guarantees collection, processing and storage of donor human milk (DHM) according to international guidelines. They are also involved in breast-feeding (BF) promotion and support.
Li *et al.*, 2024	A professional organisation that screens donors based on stringent criteria and collects, processes and dispenses DM.
Mathias *et al.*, 2023	Referred to as mothers milk bank (MMB), a facility that allows the collection and distribution of HM donated by lactating mothers other than the biological mother.
McCune *et al.*, 2021	Not provided.
Nor Azman 2024	HMB collect, screen, store, process and dispense DHM to recipient infants under the prescription of licensed healthcare professional (HCP).
Rechia *et al.*, 2016	Specialised centres that promote and encourage BF and collect, process and distribute expressed HM to infants. An objective of the HMB is to operate, in an optimised manner, the excess milk supply of each nursing mother, as well as to give advice on BF and milk donation.
Sabater *et al.*, 2014	Not provided.

### Human milk bank organisational models, funding, implementation and development

Limited information was available regarding organisational models of HMB (see Table 9). Three reviews reported that at least one HMB was hospital based^(27,28,30)^. One review discussed HMB organisational models and recommended ‘centralisation’ to improve cost efficiency, although it did not define this term or clarify its application^(26)^. Two reviews emphasised the importance of integrating HMB within both community and hospital settings at the national level to support sustainable operations^(26,35)^.

**Table 9.** HMB organisational models, funding and development information Table 9 long description.

Study	Findings
Badrul Hisham *et al.*, 2024	Review identified that sustainable HMB operations requires crucial support from the government and society (*n* 2). Emphasis on integrating HMB into national infrastructures with government support (*n* 1). Brazil has a publicly funded HMB network (*n* 1). One study advocated for ongoing BF support and measures to reduce operating costs (*n* 1). In Italy, a study at Bambino Gesu Children’s Hospital in 2019 analysed the operational costs of a HMB, identifying a significant difference in costs between collected litres in 2019 and the maximum capacity of the bank, emphasising the potential for cost reduction through increased milk collection, identifying that the highest costs are associated with salaries of medical staff and transportation. The study recommends centralisation to achieve cost savings and reduce the cost per litre of DHM (*n* 1). One study identified 5 companies, including private milk companies (PMCs), engage in the trade of HM products.
Doshmangir *et al.*, 2019	Not reported.
Dos Santos & Perrin, 2022	Single/multiple HMB, one was reported to be situation in a hospital.
Fonseca *et al.*, 2021	HMB at a university hospital (*n* 2).
Kaech *et al.*, 2022	The included studies took place in 12 HMB; located within hospitals (*n* 5), one health facility with a HM donation service and one health facility without one – India, two HMB of the PH system of the Federal District – Brazil, Mothers’ MB Northeast (MMBNE), a non-for-profit MB-USA, the HMB of a tertiary care centre in a low- and middle-income country and the HMB in a hospital, which is integrated into the Neonatology Service of the same hospital – Italy.
Kaech *et al.* 2025	Global recommendations focus on providing essential resources to incorporate HMB into breast-feeding support and neonatal care. A strong integration of HMB systems across hospitals and communities is recommended, with an emphasis on the importance of collaboration and networking among HMB, as well as the protection, promotion and support of breastfeeding, all of which are implicitly linked to sustainability. All documents recommend that the donation process be free of charge for donors. European documents report that a sustainable supply of DHM requires public and financial support from the national healthcare system. Swiss guideline discusses important elements including financing models to support HMB in Switzerland.
Kundisova *et al.*, 2019	Not reported.
Li *et al.*, 2024	Formal HMB in hospital included.
Mathias *et al.*, 2023	Not reported.
McCune *et al.*, 2023	Not reported.
Nor Azman *et al.*, 2024	Not reported.
Rechia *et al.*, 2016	Not reported.
Sabater *et al.* 2014	Two studies gave information on HMB: One was in a PH system and one in a University Hospital in Brazil.

None of the reviews reported funding sources for existing HMB. However, several emphasised the importance of cost reduction and financial support in sustaining HMB operations. One review identified increased milk collection as a potential strategy for reducing costs^(26)^, while three noted financial assistance or provision of equipment supported HM donation^(22,28,35)^. All identified policy and guideline documents recommended that milk donation remains free of charge for donors^(35)^. European policy documents further emphasised that maintaining a sustainable DHM supply depends on public and financial support from national healthcare systems^(35)^.

### Distribution or access models for donor human milk

Findings related to DHM distribution and access are presented in Table 10. Reviews consistently reported a lack of clear definitions, standards, guidelines, processes, frameworks and ethical considerations for DHM globally, alongside substantial variation in HMB practices and outcomes^(26,30)^. The need for greater clarity and standardisation in relation of HMB, protocols, frameworks and guidelines at global, national and local level was reported to support a sustainable DHM supply^(26,35)^. Only one study^(36)^, identified by McCune *et al.* ^(32)^, reported that 87 % of 86 maternity hospitals using DHM had policies specifying criteria for its use.

**Table 10.** DHM distribution or access models and how they are impacted by DHM supply, available guidelines and decision making of clinical staff Table 10 long description.

Study	Findings
Badrul Hisham *et al.*, 2024	Need for stringent procedures, and clear regulatory frameworks for HMB. Need for standardised and consistent use of informed consent to address ethical concerns (*n* 1). Global diversity in HMB practices, ranging from regulatory frameworks and commercial involvement to cost considerations and efforts to increase awareness and donation. Addressing ethical concerns, ensuring financial sustainability and promoting awareness emerge as key factors in optimising HMB globally. Lack of clear definitions, standards and ethical considerations for DHM globally, recommending further research to inform evidence-based guidance and optimal support for mothers to provide their own breast milk (*n* 1). Wide variation in the regulatory frameworks for HM globally, with most countries lacking any specific framework, and most placing HM within food legislation. Safety and ethical concerns regarding the commodification of HM are identified, underscoring the need for policymakers to address potential exploitation and prioritise BF (*n* 1). International Donor Screening: Differences in MB practices exist; self-screening tools may improve acceptance rates (*n* 1). There is a wide variability in HMB practices across Europe, including practices that could further improve the efficacy of donor HMB (*n* 1). Studies identified regulatory gaps and variability in MB practices, calling for standardisation (*n* 2). Ireland: Varied practices around DM use underscore the need for national, evidence-based guidelines (*n* 1). In US study, legislative and economic barriers restrict DM use; public policy reforms required (*n* 1). Hospitals showed diverse eligibility criteria and limited lactation support, despite emphasis on parent education (*n* 1). A US-based study (*n* 1) warns of unsafe infant feeding practices, advocating for policy reforms that enhance lactation support and access to DM. Emphasised the need for standardised, evidence-based protocols for medically indicated supplementation (*n* 1). The legal ambiguity surrounding HM in Australia identifies the need for legal definitions and a consistent network of HMB and sharing, aligning practices with established protocols for blood donation (*n* 1). Wide variation in feeding strategies, supporting the need for national HMB guidelines in China (*n* 1). Review identifies need for standardised evidence-based management and national guidelines for consistency and quality of care. HCW attitudes: NICU nurses had positive attitudes toward milk sharing (*n* 1). Health workers show a 31 % acceptance rate, with acceptance associated with knowledge on breast MBs and the health profession (*n* 1).
Doshmangir *et al.*, 2019	Not reported.
Dos Santos & Perrin, 2022	Wide range of reported donation volumes per donor; May be due to differences in HMB requirements: For example, in Brazil, there is not a minimum donation volume, in the USA, some HMB require a minimum donation of 100 ounces/3 litres.
Fonseca *et al.*, 2021	Not reported.
Kaech *et al.*, 2022	Not reported.
Kaech *et al.*, 2025	Review identified that regulation, policies and guidance are needed at the global, national and local levels to support a sustainable supply of DHM. Swiss guideline discusses important elements, such a general health policy to support HMB in Switzerland. Swiss guideline recommends limiting human milk donation to six months postpartum. It also advises providing information and instruction on the use of a breast pump, hygiene and cleaning steps to minimise wastage due to bacterial contamination. The guideline recommends taking the infant gestational age into account when recruiting donors, as well as the volume of milk excess the donors have.
Kundisova *et al.*, 2019	Not reported.
Li *et al.*, 2024	Expansion of HMB infrastructure suggested for wider access, e.g. via tertiary care hospitals and collection centres at maternity homes (*n* 1).
Mathias *et al.*, 2023	Not reported.
McCune *et al.*, 2021	87 % of level 1 maternity hospitals using DHM had policies that stated criteria for DHM, 27 % stated DHM was preferred and all required consent (*n* 1). Mother infant dyads receiving formula supplementation (*n* 376) were more likely to be non-white, publicly insured and/or non-English speaking compared to dyads who received DHM (*n* 306) – raising concerns about healthcare inequalities in DHM access (*n* 1).
Nor Azman *et al.*, 2024	Not reported.
Rechia *et al.*, 2016	Structural HMB that comply with the current legislation contribute to the achievement of high-quality services.
Sabater *et al.*, 2014	Attitude of HCP (nurses and doctors) regarding the use of donated milk; only 11 % would use donated milk (*n* 1).

Kaech *et al.* ^(35)^ reported that the location and accessibility of HMB as well as the type of transportation system used for HM influence the convenience of donations for mothers. McCune *et al.* ^(32)^ identified disparities in access to DHM noting that mother–infant dyads receiving formula supplementation were more likely to be non-White, publicly insured and/or non-English speaking compared with those who received DHM. To improve accessibility, Li *et al.* ^(28)^ recommended expanding HMB infrastructure to include tertiary care hospitals and establishing collection centres within maternity care centres.

Two reviews reported generally positive attitudes among healthcare staff towards DHM^(26,34)^, although neither examined how these attitudes affected DHM distribution or access.

### Role of key stakeholders in supporting and sustaining human milk bank

Key stakeholders in supporting and sustaining HMB include HM donors and recipients, HCP and social supports including partner, family, friends and the wider society (see Table 11).

**Table 11.** Role of key stakeholders in supporting and sustaining HMB Table 11 long description.

Study	Findings		
	HCP	Donors and recipients	Social support
Badrul Hisham *et al.*, 2024	HCP had moderately influential role in BM donation. Barriers to milk donation: post-discharge processes, lack of awareness and insufficient support from clinic staff (*n* 1). Need to improve their knowledge and attitudes through education for community acceptability (*n* 1). A survey in India underscores the importance of providing knowledge to antenatal mothers and the role of nurse practitioners (NP) in enhancing awareness (*n* 1). Studies in the Northeast US hospitals identify the importance of parent education, while emphasising limited lactation support during DHM provision (*n* 1).	Motivations of donors/positive attitude towards donation: altruism; confidence in milk supply; confidence in professionalism; social support; education; income; birth type; BF experience; encouragement to donate; knowledge; helping bereaved mothers cope with loss and reconstruct their identities as mothers (*n* 1). Barriers to donating: religious and cultural beliefs including potential rejection of DM among Muslim families due to milk kinship and donor anonymity; concerns about safety and HIV screening; post-discharge processes; lack of awareness and/or knowledge; lack of support from HCP; expense; impact on mother–child bonding. Acceptability of DHM: pregnant women exhibit a 61.5 % acceptability of donated breast milk, influenced by higher education, being Muslim, awareness of donated MB and the presence of a serious medical condition (*n* 1). Postpartum women display awareness and acceptance disparities influenced by educational levels, nationality, and NICU admission (*n* 1).	Substantial social support facilitated donation (*n* 1). Cultural dimensions and religious concerns: Muslim families concerned due to milk kinship and donor anonymity (*n* 5). Suggested public awareness campaigns to address resistance among all family members and foster interactions between donors and recipients and increase donation (*n* 2). Positive experiences with informal milk sharing shaped by faith, mistrust of medical systems and sacred traditions among Orthodox Jewish Communities (*n* 1).
Doshmangir *et al.*, 2019	Facilitators of donation: encouragement from HCP can be a major reason for donating HM, as donors come to know of the need for DM and feel that it is a social responsibility. Verbal encouragement to donate (*n* 1); support from the MBs for the donor (*n* 1); benefits for donors (*n* 1); provide guidance on appropriate procedures for collection and storage of milk at home (*n* 1); provision of information about donating during pregnancy/prenatal care (*n* 2); visits of donors to the HMB to understand the process of pasteurisation (*n* 2); health staff and professionals (*n* 7); supporting bereaved mothers in their lactation needs (*n* 2). Barriers to donation: lack of knowledge of other professionals (*n* 1); lack of support from health professionals (*n* 1).	Facilitators: a strong belief in the value of BF (*n* 2); helping and connecting (*n* 3); having excess milk (*n* 13); knowledge of the benefits of mother milk for preterm infants (*n* 1); sense of satisfaction gained from helping a child (*n* 2); understanding the needs of the babies (*n* 2); (7) social involvement18 (*n* 1); having a history of milk donations (*n* 1); Knowing the needs of the HMB (*n* 1); hoping someone else will do the same for them if needed (*n* 4); having a positive attitude toward milk donation (*n* 1); avoiding waste (*n* 2); feeling of motherhood following a perinatal loss (*n* 2); finding meaning in and integrating the experience of perinatal loss (*n* 1); a way for bereaved mothers to grieve their loss14 (*n* 1); pumping milk to stimulate lactation (*n* 1); having self-confidence (*n* 2); financial inducement (*n* 1). Barriers: the need for the donor to plan for donating to the HMB (*n* 1); anonymity of the identities of donors and recipients (*n* 2); not qualifying for milk donation (*n* 2); milk pasteurisation (*n* 2); willingness to help mothers and children that are not considered qualified by the HMB (*n* 1); pumping inconvenience (*n* 2); donating milk through unofficial channels (*n* 2); reduction of milk (*n* 2). Absence of personal connection in donating milk to a MB (*n* 1); lack of knowledge about MB (*n* 5); misconceptions about the costs of MBs (*n* 2); not knowing whether the MB is for profit or nonprofit (*n* 1); misinformation (*n* 1).	Social facilitators: friends (*n* 3); family (*n* 5); sources of information regarding milk exchange (*n* 2); media (*n* 4); advertising (*n* 1); education (*n* 5); social values (*n* 1); society (*n* 3); local culture (*n* 1). Social barriers: resistance to creation of Western-style MBs (*n* 1); religious beliefs (*n* 9); cultural beliefs (*n* 2); incomprehension at work (*n* 1).
Dos Santos & Perrin, 2022	Enabler of donation: encouragement to donate and receiving information about MBs from HCP. HCP major source of information in Brazil.	Reasons for donation: altruism; excess milk; avoiding waste; Identity as donor had a special meaning for bereaved mothers (*n* 3). Barriers for donation: finding time to pump; reduced milk production; limited information provided prenatally; returning to work; distance from MB; no support at work (*n* 5). Milk Volume and Type: A wide range of reported donation volumes per donor, attributed to differences in MB requirements (*n* 10). In India and Spain, donors with infants in the NICU/hospitalised provided significantly higher volumes than donors without hospitalised infants (*n* 2). Donor type was mostly first time (v. repeat) in all regions (*n* 5). Mature milk (after 1 month postpartum) commonly donated (*n* 8). As a result, DHM is likely lower in protein than the colostrum and transition milk. Limited information about donation duration (range 2 weeks to 13 months) (*n* 4).	Online sources were reported as major sources of information in Korea and China (*n* 5).
Fonseca *et al.*, 2021	Important that HCP counsel pregnant women about HMB and the possibility of donation. Most common motive reported by donors was ‘encouragement by a health professional’ (61.3 %) (*n* 1). HCP play an important role in training mothers/babies to BF and in promoting skin-to-skin contact and exclusive on-demand BF. HCP should be trained to help recruit donors. HMB staff and professionals involved in maternal and infant care should receive routine training to meet needs of mothers and infants, particularly those related to initial BF difficulties. BF support provided by HMB health professionals includes helping mothers whose babies have been admitted to a NICU stimulate milk flow until the baby is able to suck at the breast and be discharged and exclusively breastfed (*n* 1).	Facilitators to donation: Receiving Information on BF; support expressing breast milk, stimulating milk production and preventing breast engorgement; Knowledge of importance of donation early postpartum. Barriers to donation: uncomfortable and/or scary environment. Socio demographic characteristics of donors: Completed secondary education; aged between 20 and 29 years; having steady employment (*n* 1).	Not reported.
Kaech *et al.*, 2022	Receiving support from HCP positively impacted women’s willingness to continue donating. A shortage of human resources (including healthcare professionals) negatively affected the volume of donations (*n* 1).	Duration. The volume of milk donated increased with the duration of the HM donation (*n* 2). Women who began donating their milk earlier (before four months postpartum) tended to donate larger volumes than women who first donated after four months postpartum (*n* 1). Infants. Women with preterm infants donated larger volumes of DHM than those with term infants (*n* 2). Admission of neonates to the NICU was associated with larger donation volumes by mothers. Mothers of infants with a low birthweight donated more DHM than mothers with higher birthweight infants (*n* 1). Mothers who reported that their infants had thrush donated more milk than those who did not. BF their own child potentially has a positive or negatively impact the frequency of donations (*n* 1). Baby’s growth was self-reported by some donors as potentially having a negative influence on the frequency of milk extraction and production. Women with 4–7 pregnancies have a higher likelihood of donation recurrence than those with between 1 and 3 pregnancies. Women with a higher education level were twice as likely to become regular donors (*n* 1). Maternal age was both positively (*n* 1) and negatively (*n* 1) associated with the volume of donation. Women who were unemployed, homemakers, or workers donated significantly smaller volumes of milk than women in other professional categories. Previous milk donors donated greater volumes than first-time donors (*n* 1). Motivation. Having an excess of milk; needing to pump to stimulate lactation donated significantly more than those who disagreed with that statement. Donation Sustainability decreases: Birthweight; Growth of the baby; Mother ‘routines (going out, contraceptive use, return to work); Experience of BF simultaneously; Physical fatigue; Presence of negative emotions (all *n* 1); Variation in Donation Sustainability; Baby feeding frequency (*n* 1); Maternal age (*n* 2); Profession (*n* 1); Self-hydration (*n* 1);Diet (*n* 1); Donor’s availability of time (*n* 1); Donation Sustainability increases: Frequency of milk expression (*n* 1); Nothing interferes with milk production (*n* 1); Time of day for expressing milk (*n* 1); Family support for donation (*n* 1); Previous milk donation history (*n* 1); Motivated to donate by having an excess of milk (*n* 1); Motivated to donate by having to pump to stimulate lactation (*n* 1); Duration of donation (*n* 2); Start of donation (*n* 1); Preterm infant (gestational age) (*n* 3); Admission to NICU (*n* 1); Thrush in the infant (*n* 1); Number of pregnancies (*n* 1); Education level (*n* 1)	Receiving support/lack of support from their families positively/negatively impacted women’s willingness to continue donating (*n* 1).
Kaech *et al.* 2025	European documents identified the need for training HCP who recruit donors (*n* 2). Global documents stress the importance of promoting and protecting exclusive breastfeeding to raise awareness and allow for a larger donor pool. Lactation education and support are proposed to help minimise barriers to donation and to support mothers in building their milk supplies. Recommended ongoing quality, training of HCPs and updating of knowledge.	Global documents: Recommendation to target bereaved mothers or donors who have a surplus of milk beyond their own infants’ needs. However, no specific characteristics of human milk donors were discussed at the social level. European docs: At the micro level, no elements explicitly associated with the sustainability of donation were related to donors or their infants. Implicitly related to sustainability donors should have an excess of milk, that most donors’ infants received DHM while being hospitalised in the neonatal intensive care unit and that human milk donors may donate as an altruistic gesture following their own infants’ use of DHM. Factors in recruiting donors: mothers whose children are premature, those with surplus breast milk and bereaved mothers.	HM donor recruitment communication methods: written documents, referrals (e.g. during routine care, antenatal classes, support groups), mass media and the donors themselves (talking with families, peer support groups, and the community). The engagement of HMB, local leaders and the community can facilitate the recruitment of donors. In European documents: At the meso level: (i) the importance of raising public awareness about donation and education at the local and national levels to facilitate donor recruitment through an adequate public-awareness strategy, (ii) effective recruitment systems (using various channels) (iii) recruit more donors through public campaigns focusing on altruism and solidarity, among others, to increase the size and diversity of the donor pool.
Kundisova *et al.*, 2019	HCPs are important source of information (*n* 3). They should be involved in identification and recruitment of new donors given their contact with future mothers during routine pregnancy appointments. 26 % of donors were primarily influenced by a HCP (*n* 1). 44 % of donors reported that they did not receive any information regarding the possibility of donating, before their baby was born (*n* 1); 50 % of donors received information about donation during their stay in the hospital; Higher rates of information among donors in comparison to non-donors (91 % *v*. 66 %); the principal source of information was HCPs (73 % *v*. 69 %) (*n* 1). Recommendation from HCPs and knowledge of infant needs were important motivations among Brazilian donors (*n* 2). The support of HCPs is necessary to promote BF initiation and maintenance (*n* 2).	Motivation donors: positive emotion, excessive milk production and the desire to help to preterm babies in need (*n* 3); knowing the needs of HMB (*n* 1). Barrier: Doubts about what happened with milk after donation and the identity of the recipients (*n* 1).	20 % of donors received information from family members and friends, 7 % by a mass media campaign (*n* 2). Higher rates of information among donors *v*. non-donors (91 % *v*. 66 %), Studies reported insufficient information is available by mass media (*n* 1), although targeted campaigns could raise interest in milk donation; The involvement of former donors in the recruitment process is likely to be an effective method for recruiting new donors (*n* 1).
Li *et al.*, 2024	HCPs could take on a pivotal role in informing potential donors and recipients. The limited awareness about formal milk donation restricts mothers’ options and access to resources. Women’s views on HM sharing are shaped by both HMB-associated and other HCPs. Staff in HMB had the most frequent contact with donor and recipient mothers. A mother’s trust in HCPs frequently sways her decision to accept donations. Women see HCPs as the key influencers and trustworthy sources. HCPs should receive training to increase the initiation and continuation of milk donation. Providing on-the-job training for HCWs who are not affiliated with HMB may ensure that potential donors or recipients are informed about milk sharing during medical consultations. Education for mothers, family and community members, acknowledgement during the early antenatal period, explanation of infection control procedures, and DM strategies are suggested.	Facilitators of donation: Psychological satisfaction, both in their role as a mother (*n* 4) and as a donor (*n* 2); Altruism (*n* 7). Reduce waste of surplus milk (*n* 7); Comforting grief ritual for bereaved mothers (*n* 5); Imbue the life of the deceased child with profound meaning, foster a sense of life continuity, and aid in reconstructing a mother’s maternal identity (*n* 2); HMB personnel suggest that remote work allow parents to have less anxiety about keeping extra milk (*n* 1). Barriers to donate: fear of insufficient milk for own baby (*n* 2); investment of time and money in the process; travelling to and from the HMB; organising childcare; conducting body checks and tests, pumping milk, cleaning bottles and maintaining sterile conditions is a laborious process; fear of potential disease transmission or milk (*n* 5). Recipients’ barriers: Feel guilty/jealous about not being able to feed their children themselves. Witnessing their children thriving on the donated milk often leads to later acceptance. Investment of time and money in the process. Concerns about disease transmission. Others’ milk seen to threaten the sacred one-on-one mother-children bond and maternal competence that breastmilk embodied (*n* 6). Concern that BM may transmit traits from the mother (*n* 3). Significant amount of donors reported feelings of discomfort about receiving donated milk (*n* 1).	Family members/relatives impacted women’s decision to donate-positively or negatively, depending on their support. Community values and religion can shape perception of HM sharing and whether it is acceptable (*n* 5). Information sources: Internet, social media, radio, and newspapers (*n* 5).
Mathias *et al.*, 2023	Barriers to donating: Lack of practical and psychological support from the health professionals (*n* 3), shortage of trained staff (*n* 1), issues in communication with staff (*n* 1). Facilitators to donating: Encouragement from HCPs (*n* 1) trust in HCPs (*n* 1), counselling by paediatrician/lactation counsellor during well-baby clinic (*n* 2) and support from community health workers enhance the knowledge levels about MB (*n* 1). Barriers to accept: Negative feedback or lack of support from HCP. Facilitator to accept support from HCPs (*n* 5).	Barriers to donation: limited knowledge; limited time due to daily chores; physical and psychological stress from pumping, freezing, or storing milk; cost and distance to the MB; personal dislike; difficult expressing BM; lack of time; insufficient milk; hygiene concerns; medical condition of the mother (e.g. engorgement, cracked or chapped nipples and breast infection); risk of disease transmission; fear of transfer of genetic traits and familial diseases; negative influence affecting the mother–child relationship; mother unable to meet the needs of her own child; lack of appropriate storage arrangements; existing practices and perceptions; concerns about process of becoming a donor (i.e. screening); changes in skin colour if the milk is received from another mother (*n* 1); the possibility of fluctuation in breast size; concern about impact on recipient mother; Dealing with milk donation rules and restrictions. Facilitators to donate: Desire to help infants (*n* 5), personal values such as security, tolerance, self-direction, and social concern (*n* 5), awareness about breastmilk donation (*n* 4); Excess milk supply (*n* 2); economic compensation, reward or appreciation; own baby being healthy and growing well; stimulate lactation; knowledge of the shortage of breastmilk (*n* 1 for all). Barriers to accept DHM: Fear of disease/allergy transmission; fear transfer of genetic traits (*n* 4). Concerns about milk quality/safety, maintaining the cold chain, and transporting donated milk (*n* 3); perceived hygiene of the donor/donation process (*n* 5); belief that babies had to be fed by their mother (*n* 1); negative influence on bonding between mother and child (*n* 5); supply could not keep up with demand (*n* 1); lack of awareness/knowledge about MBs (*n* 1); concern about insufficient milk (*n* 1); lack of trust in people/donated HM (*n* 2); children not meeting requirement criteria (*n* 1); travel and cost (*n* 2); concern about the identity (religion/health) of the donor (*n* 2); lack of paid parental leave policy in the USA mean that time and effort involved conflicted with mothers’ need to return to work (*n* 1). Facilitators to accept DHM: logical solution for insufficient milk/preterm baby/ LBW baby in NICU (*n* 6); illness of mother/on medication (*n* 4); screening health status of donor’s nursling (*n* 1); unavailability of mother to BF (*n* 1); lack of trust in infant formula (*n* 1); child unable to tolerate formula feed (*n* 1); awareness about the importance of BM (*n* 1) and donated HM (*n* 1); child not gaining weight (*n* 2); lack of informed decision-making and transparency (with respect to personal and medical history) (*n* 2); wet nursing only by family member (*n* 2); newborn unable to suck or is sick (*n* 3) and families with orphaned or abandoned child (*n* 1).	Social facilitators for Donation: Family; Support from the spouse/partner i.e. physical help with sterilising equipment and emotional support (*n* 1); community approval and support (*n* 2). Social barriers for Donation: Family socio-cultural values (*n* 1); husbands’ decision-making power (*n* 1); Cultural and religious, practices and taboos the community regarding breast milk donation creating barriers across countries (Kenya, South Africa, Eastern Uganda, Turkey, and India) (*n* 6); Poor education within families (*n* 1); Limited familial support (*n* 2). Social barriers for Accepting: Lack of community or family support (*n* 2); husbands’ decision-making power (*n* 1); family customs and beliefs and lack of knowledge/awareness (*n* 3); Unacceptable religious and cultural practices(*n* 2); Social facilitators for Accepting: Strong spousal/partner support; the suggestion by a friend or relative; community knowledge (through education) and support (*n* 1), community leaders and traditional healers support; Considering screening to involve testing heritage and genes (culture compatibility) (*n* 1); perceiving that receiving milk did not lead to religious and ethical problems (*n* 1).
McCune *et al.*, 2021	Nurses lacked knowledge of DHM, presented logistical concern for implementing a DHM policy in a well-baby nursery (*n* 1).	Recipients: 58 % of mothers preferred DHM supplementation over formula (*n* 1). Mother–infant dyads receiving formula supplementation (*n* 376) were more likely to be non-white, publicly insured and/or non-English speaking compared with dyads who received DHM (*n* 306)- raising concerns about healthcare inequalities in DHM access (*n* 1). Knowledge of DHM: Maternal concerns related to safety of DHM, and lack of information reported (*n* 1). Women reported that formula is familiar and DHM is not, DHM is costly and logistically challenging.	Not reported.
Nor Azman *et al.*, 2024	HCP were the most common source of information about donated breast milk (*n* 1). HCP were found to be the source of knowledge for only 13.1 % of the participants (*n* 1). Lactation consultations helped participants gain awareness of MB (*n* 3).	Knowledge of HMB: 67.7 % to 95.1 % of Muslim women were not aware of HMB (*n* 6). Differences in study setting (urbanisation and size) impacted level of knowledge reported, i.e. in Iran 12.8 % of women knew of HMB (*n* 1) and in Turkey 62.5 % did (*n* 1). Perceptions of breast milk donation 28.7 % supported the idea of HMB for infant health, while 20.9 % supported it for emergency cases (*n* 1). 51.8 % of Muslim women were unwilling to donate their breast milk-religious concerns as the primary reason for refusal. Potential issues arising from the marriage of individuals who have been breastfed by the same person were also cited as a significant factor. Muslim women believed that the breast milk donation process posed a significant danger of transmitting infectious diseases to recipients (*n* 1).	Partner approval shaped opinions of women considering donating or accepting donor breast milk (*n* 1). In Islam, a woman must obtain her husband’s permission before deciding to feed breast milk to another person’s infant, milk kinship is permissible and the milk father needs to understand his responsibilities to avoid any future complications (*n* 2). Information sources: 42.4 % of participants knew about MBs (*n* 1) Most common source of information were friends, relatives, internet, followed by TV (*n* 6).
Rechia *et al.*, 2016	HCPs provided most information and/or guidance on BF and milk donation (*n* 2). Health professionals are responsible for guiding nursing mothers on the importance of BF and milk donation, and encouraging them to volunteer for donating breast milk, especially during pre-natal, delivery and postpartum (*n* 3). A need to improve health education in maternity units, particularly in joint accommodation in these units and in neonatal care units; greater attention should be given to guidance on breast milk donation by health professionals, associated with guidance on BF (*n* 2). Decision-making regarding milk donation is directly influenced by the assistance received by nursing mothers at the HMB (*n* 1). Valuation of milk donation and appropriate communication between professionals and nursing mothers are key enablers of donation (*n* 3). The number of donors would significantly increase if guidance on milk donation began in the prenatal period (*n* 3). A lack of well-prepared health professionals to provide effective guidance to nursing mothers detected. Health education actions targeted to the promotion, protection and support to BF and to the appropriated training of health professionals who provide care to pregnant women or nursing mothers who seek these services may result in the increase in the number of HM donation. Support and encouragement from HCP are essential for the mother and the infant (*n* 1). A manual with guidance on home nursing consultation, allowing its systematisation resulted in improvement of health professional/nursing mother relationship and in a more effective promotion of BF and milk donation.	Motivators; engorgement (*n* 5), excess milk supply (*n* 3), knowledge of the importance of breast milk for hospitalised infants (*n* 5), altruism (*n* 6) and previous experience of BF difficulties (*n* 2). Barriers: lack of adequate information (*n* 2), lack of time and reduced milk supply because of their work activities (*n* 3) and number of children (*n* 1). Adolescent milk donors faced unfavourable socioeconomic and perinatal conditions, requiring special care in BF management (*n* 1).	Not reported.
Sabater *et al.*, 2014	Healthcare professionals (HCP) role in the recruitment of first-time donors; donors were motivated by HCP recommendation (*n* 1), identified the need to involve HCP in the promotion of donation including prenatal visits, training and encouraging mothers about the process and encouraging donations with the help of volunteers and the provision of technical means.	Motivators to donate: majority had positive experiences with milk donation, will donate again if possible; excess milk production; donating helped with engorgement; altruism or being charitable; maintaining the duration of BF; contact with the activities of the MB; knowledge of MB, including procedures/knowing the needs of MB/ hope that someone would do it for them if they needed; problems with BF, i.e. mastitis. Barriers to donation: extraction difficulties and lack of information.	Social support, particularly from partners and family, important for initiation and maintenance of donation. Donors can give support and attract other donors in their environment disseminating information and motivation.

DHM donors and recipients play a key role in sustaining HMB. Common factors that facilitated donation were excess milk production or engorgement (*n* 10)^(21–23,26,27,30,31,33–35)^; altruism/helping (*n* 9)^(21,22,26,28,30,31,33–35)^; stimulating lactation (*n* 4)^(22,27,31,34)^; knowledge/education/belief in HMB importance, needs and processes (*n* 7)^(21,22,26,27,31,33,34)^; hope that someone would do it for them if they needed access to DHM (*n* 2)^(22,34)^; positive emotion (*n* 3)^(21,22,28)^; experience of BF and/or BF difficulties (*n* 3)^(26,33,34)^; having a history of milk donations (*n* 2)^(22,23)^; avoiding waste (*n* 4)^(22,26,28,30)^; finding meaning, identity and grieving following a perinatal loss (*n* 4)^(22,26,28,30)^ and economic compensation, reward or appreciation (*n* 2)^(22,31)^. Other facilitators were self-confidence (*n* 1)^(22)^; remote working (*n* 1)^(28)^; own baby being healthy and growing well (*n* 1)^(31)^. Global policy and guideline documents recommended that donor recruitment include bereaved mothers and those who have a surplus of milk beyond their own infants’ needs (*n* 1)^(35)^.

Frequently identified barriers to donation were lack of information, knowledge, awareness or misconceptions about HMB (*n* 8)^(21,22,26,29–31,33,34)^. Other commonly reported barriers were lack of time or financial resources to engage in donation activities (*n* 6)^(22,26,28,30,31,33)^; concerns about infection or disease transmission (*n* 4)^(26,28,29,31)^; difficulties with milk expressing or inconvenience of pumping (*n* 3)^(22,31,34)^ and reduced or insufficient milk supply (*n* 3)^(22,28,30)^. Additional barriers included absence of a personal connection to, or discomfort with donating to a milk bank (*n* 2)^(22,31)^; concern regarding the impact on mother–child bonding (*n* 2)^(26,31)^ and religious or cultural beliefs (*n* 2)^(26,31)^. Barriers identified by single reviews included unfavourable socio-economic or perinatal conditions^(33)^; lack of affordable transport^(31)^; ineligibility for donation^(22)^; milk donation through unofficial channels^(22)^ and maternal medical condition^(31)^ (all *n* 1).

Four reviews reported facilitators and barriers to recipients’ acceptance of DHM from HMB. Facilitators included viewing DHM as a logical solution for infants requiring HM for various reasons (*n* 2)^(26,31)^ when the mother was unable or unavailable to BF (*n* 2)^(26,31)^ and awareness of the importance of DHM (*n* 2)^(26,31)^. Additional facilitators were lack of trust in or infant intolerance to formula (*n* 1)^(31)^ and observing positive infant outcomes after receiving DHM (*n* 1)^(32)^.

Reported barriers included maternal concerns regarding the safety and quality of DHM during transportation and insufficient information (*n* 3)^(28,31,32)^; financial or logistical challenges (*n* 3)^(28,31,32)^ and perceptions that using another mothers’ milk could threaten the maternal–infant bond (*n* 3)^(26,28,31)^. Other barriers, each identified in a single review, included feelings of guilt or jealousy over not breast-feeding personally (*n* 1)^(32)^, lack of trust in donated milk (*n* 1)^(31)^, infants not meeting eligibility criteria (*n* 1)^(31)^ and concerns about the donor’s identity, including religion or health status (*n* 1)^(31)^.

HCP were identified as key stakeholders in supporting and sustaining HMB networks across all thirteen reviews. They played a key role in facilitating donation, including identifying, recruiting and motivating first time donors (*n* 6)^(21,22,27,28,30,34)^. Support from HCP positively influenced women’s willingness to continue donating (*n* 2)^(21,23)^, whereas one review reported that a shortage of HCP negatively affected donation volume^(23)^. HCP were also recognised as important sources of information, knowledge and guidance for donors and potential donors (*n* 7),^(21,22,28–31,33)^ while lack of HCP support was identified as a barrier to donation (*n* 3)^(22,26,27)^. Several reviews reported insufficient training, resources or knowledge among HCP and recommended enhanced education to strengthen their role (*n* 6)^(26,28,31–33,35)^. In addition, HCP were noted to play a key role in promoting the acceptance of DHM among recipients. One review identified support from HCP as the most influential facilitator of DHM acceptance, while negative feedback or absence of such support was found to hinder acceptance^(31)^.

Seven reviews identified social support as a key factor in facilitating and sustaining milk donation to HMB. Sources of support included partners, family members, friends and other donors, all of whom played important roles in donation initiation and maintenance (*n* 8)^(21–23,28,29,31,34,35)^. Common sources of information about HMB were friends, relatives, the internet, social media and mass media (*n* 5),^(21,22,28–30)^ while one review also noted healthcare providers and HMB leaders as information sources^(35)^. Limited mass media information was reported in one^(21)^. Two reviews emphasised the need to raise public awareness and enhance donor recruitment through targeted communication strategies^(21,35)^.

Cultural and religious beliefs, along with lack of social support, were cited as barriers to donation (*n* 3)^(22,28,31)^. Similar social factors influenced acceptance of DHM; facilitators included support from partners, family and friends (*n* 2)^(29,31)^; community knowledge and endorsement (*n* 1)^(31)^ and perceptions that receiving milk did not pose religious or ethical concerns (*n* 1)^(31)^. Conversely, social barriers included limited family or community support (*n* 2)^(29,31)^, lack of family knowledge or awareness (*n* 2)^(29,31)^ and perceptions of milk sharing as culturally or religiously as unacceptable (*n* 2)^(29,31)^.

### Facilitators and barriers to the development, implementation and sustainability of human milk bank

Facilitators and barriers to HMB development, implementation and sustainability are summarised in Table 12. Factors facilitating HMB establishment included the nutrient-rich content and cost-effectiveness of DHM, the prevention of milk wastage and recognition of the need for HMB (*n* 1)^(26)^. To address religious concerns, Doshmangir *et al.* ^(22)^ proposed that HMB’s serving Muslim populations ensure donors and recipients are known to each other, aligning with religious principles of milk kinship. Explaining HMB processes to recipients was also identified as a facilitator of DHM acceptance (*n* 1)^(31)^.

**Table 12.** Facilitator and barriers to development, implementation and sustainability of HMB Table 12 long description.

Study	Findings
Badrul Hisham *et al.*, 2024	Findings emphasise the critical need for appropriate information, education, and counselling to promote the adoption and establishment of HMB in Japan (*n* 1). A study in Brazil identified ambiguities around BF and the establishment of HMB, emphasising historical processes and discussing the cultural script surrounding BF. A study in Ghana assesses readiness for a HMB, with concerns including perceptions of HMB as strange, fear of infections, religious beliefs, and insufficient information (*n* 1). Germany, Austria, Switzerland: Low usage rates of DM noted, though hospitals showed interest in uptake if DM were available (*n* 1). Ghana: Informal milk sharing exists; Need for educational campaigns about risks and potential of HMB (*n* 1). South Africa: Barriers include HIV fears, cultural beliefs, and safety concerns, calling for targeted educational strategies (*n* 1). Identified role of technical advancement in maintaining quality, reducing waste and enhancing sustainability in HMB: the REAMIT project introduced Internet of Things sensors and big data to monitor milk quality during transportation. Studies identified the importance of donor screening processes, donor selection impact.
Doshmangir *et al.*, 2019	Obstacles to establishing HMB in Muslim countries. Some mothers refuse to donate their milk due to religious concerns, and some families avoid receiving donated milk. A culturally accepted approach proposed is that HMB in Muslim countries should require the donor and the receiver to know each other’s identity. In one study, 36.3 % of women expressed some kind of religious concerns, and 28.9 % believed that it would lead to social and moral problems. Turkish people support a HMB that follows a single-donor policy-the child received milk from a single donor, and both the donor and the receiver knowing each other’s identity. In Ethiopia with limited information and misconceptions about the safety of BM, the acceptance of donation and receipt of DHM is very low. Systemic barriers: Lack of health care centres as a source of information about milk exchange (*n* 1); The HMB time consuming processes (*n* 4); lack of HMB or lack of access to the HMB (*n* 4). Systemic facilitators: provide guidance on appropriate procedures for collection and storage of milk at home (*n* 1); financial support (*n* 1): provision of information about donating during pregnancy/prenatal care (*n* 2); support from the milk banks for the donor (*n* 1).
Dos Santos & Perrin, 2022	Not reported.
Fonseca *et al.*, 2021	Not reported
Kaech *et al.*, 2022	Barrier to continued donation (sustainability): distance to the HMB (in the absence of HM collection or transportation services) (*n* 1); shortage of human resources in HMB (including HCPs (*n* 1); lack of support at their workplace (e.g. lack of emotional support or lack of encouragement to donate milk or BF their own child) (*n* 1).
Kaech *et al.,* 2025	One document identified a need for legislation and regulation of HM and HMB. Swiss guidelines discuss the need for a legal framework for DHM and general health policy to support HMB in Switzerland. Another recommendation was to minimise the burden of the screening process for donors. Regarding the human milk bank location, facilities (space) and the services offered, several recommendations explicitly linked to the idea of a sustainable supply of DHM were made to render the donation process as convenient as possible. For instance, the location of the human milk bank (its accessibility) and a human milk transportation system can have an impact on convenience for human milk donors. Spatial resources, such as lactation support rooms and/ or private rooms for breast-feeding and milk expression, were recommended for donors’ comfort. Other elements, such as supporting donors and providing milk collection supplies, are identified.
Kundisova *et al.*, 2019	Donors wanted to receive more support from the HMB, and to be adequately informed about the HMB and the possibility of donating before giving birth to their baby (*n* 1). Enhancing donor recruitment and sharing information with potential donors is a priority for HMB (*n* 1).
Li *et al.*, 2024	Compensation to promote HM donation: Studies argues that financial compensation acknowledged the monetary value of the labour involved in donating breast milk and may encourage more donations and benefit donors financially (*n* 1). However, others argued that such incentives may undermine the quality of health and moral value of the practice and potentially exploit the economically vulnerable donors (*n* 2). Agreement that equipment be supplied/rented to donors for free, despite concerns on feasibility (*n* 2). Studies showed that such assistance from HMB, such as pick-up service can make donation more acceptable (*n* 1). Systemic barrier towards milk donation identified: exclusion of bereaved mothers in donation programmes. Staff shortages reduced milk collection (*n* 1).
Mathias *et al.*, 2023	To establish HMB, measures should focus on reducing delays; reducing the time spent at the donation centre, providing pick-up service to donors, educating the community about breast milk donation and encouraging male partners to participate could all help to boost acceptance of breast milk donation. BF donation and acceptance rates may be increased by establishing laws to govern the HMB and the marketing of formula foods. Barriers to donation: improper acquisition and storage process of breastmilk (*n* 2), limited infrastructure, technical and financial support in HMB (*n* 1), hospital discouraging donation due to risk of infection (*n* 1), dealing with milk donation rules and restrictions in the North American region (*n* 1). Limited affordable transportation to the HMB (*n* 1); staff shortages (*n* 1). Barriers accept DHM: Hospital/profession policy that discouraged the usage of donated milk (*n* 1). Facilitators to accept DHM: testing and sterilising equipment processes (*n* 2), explaining the process of screening, pasteurisation and HMB: affirming that donors were healthy (*n* 1).
McCune *et al.* 2021	Not reported
Nor Azman *et al.*, 2024	7/12 publications examined contained information on the opinions of Muslim women re. HMB. These studies discovered that anywhere from 25.7 % to 76.7 % of Muslim women in Turkey did not approve of a HMB project (*n* 5). Primary reason due to religious beliefs, specifically concerns about the possibility of marriage between individuals who have consumed milk from the same donor (*n* 5). A study conducted among 303 Muslim women found that 47.1 % of them supported the establishment of a HMB, 25.7 % were against it, and 27.3 % stated that they did not have complete knowledge of the issue (*n* 1). The main reason for objection identified the possibility of problems regarding marriage in the future. 76.7 % of the participants who did not want a HMB to be established stated that they did not want it because of the risk of disease transmission (*n* 1). The same study identified several significant factors that act as barriers to HMB establishment, including lack of support from husbands, religious concerns, commercial gain, hygiene and decreased nutritional value (*n* 1). Out of seven studies, only one study showed that a significant number of Muslim women (77.5 %) support the establishment of MBs in Turkey (*n* 1). Another study found that 41.5 % of women who agreed with the establishment of a MB also indicated that they would be willing to use the service (*n* 1). Factors that facilitate the establishment of HMB include nutrient-rich content, cost-effectiveness, avoiding wastage and the need for a HMB (*n* 1).
Rechia *et al.*, 2016	Structural factors that interfered with the donation of HM were: Inappropriate physical structure of facilities; incomplete records of pasteurisation of HM; poor hygiene of breasts and hands; equipment insufficient to meet the demands; understaffed personnel. Studies identified the need to improve technical support, assistance and monitoring of donors (*n* 2).
Sabater *et al.*, 2014	Institutional care, encouragement and communication about the donation process was a factor in donors’ decision to donate (*n* 1).

System-level facilitators of milk donation included institutional support and the provision of clear information and guidance about the donation process (*n* 5)^(21,22,28,34,35)^, as well as adequate technical support in HMB operations (*n* 2)^(26,31)^. Mathias^(31)^ emphasised the importance of reducing donor time at collection centres, offering milk pick-up services and involving male partners in the donation process. Two reviews identified the need for appropriate, education, counselling and information dissemination to promote HMB,^(26,31)^ while others underscored the necessity of legal frameworks to govern HMB operations^(31,35)^.

Among Muslim women, barriers to HMB development and implementation included religious concerns about disease transmission and lack of spousal support (*n* 3)^(22,26,29)^. Additional concerns involved perceptions of HMB as unfamiliar or profit-driven institutions (*n* 2)^(26,29)^. Misconceptions and insufficient information regarding the safety of DHM also contributed to lower acceptance (*n* 2)^(22,26)^, although one study reported support for HMB establishment among Muslim women^(26)^.

Structural factors identified across five reviews included inadequate physical facilities (*n* 1)^(33)^, incomplete pasteurisation records of HM (*n* 1)^(33)^, poor hygiene practices among donors (*n* 1)^(33)^, inefficient equipment and processes (*n* 2)^(31,33)^ and shortage of human resources including HCP (*n* 4)^(23,28,31,33)^. Additional challenges were distance to or limited access to HMB (*n* 3)^(22,23,31)^, lack of workplace support, (*n* 1)^(23)^ and time-consuming HMB processes (*n* 1)^(22)^. System-level recommendations from policy and guideline documents identified in Kaech *et al.* ^(35)^ included minimising the burden donor screening and ensuring convenient HMB locations, facilities and operational systems.

## Discussion

This umbrella review revealed an understanding of the purpose and functions of HMB but also highlighted substantial gaps in their practical, structural and financial frameworks. No clear consensus was identified regarding the defining attributes of a HMB, although several core functions were consistently reported. HMB are primarily responsible for donor recruitment and selection, milk collection, screening, treatment processing, storage and distribution or dispensing of DHM. Notably, several reviews identified an expanded role for HMB in promoting, supporting and encouraging breast-feeding^(21,22,27,33,35)^.

No information on funding sources was reported. Limited detail was also available on HMB physical or organisational structures. ‘Centralisation’ of HMB was identified as a recommended organisational model to achieve cost savings and promote national level integration for long-term sustainability; however, the term was not defined within the included reviews^(26)^. HMB were described as operating within a variety of settings, including public and private hospitals, and private milk companies. Two reviews identified the need to develop nationally coordinated HMB systems to safeguard sustainability, while six reviews did not report organisational models^(21,22,29,31–33)^. It was recommended that a sustainable supply of DHM requires both public and financial support from national healthcare systems.

Globally, HMB lack clear definitions, standards and ethical frameworks, particularly in relation to DHM distribution and access models. Considerable variability and limited standardisation were observed across HMB practices, protocols, frameworks and guidelines. Several reviews reported the need for harmonised guidance at global, national and local levels to support sustainable DHM supply. Only one review reported the existence of policies specifying criteria for DHM use^(32)^. However, these were limited to individual maternity hospitals, with no evidence of equivalent policies at national or global levels. Notably, six reviews did not report items in this area^(21–23,27,29,31)^. This lack of consistency underscores the need for comprehensive, standardised frameworks to ensure the sustainable provision of DHM worldwide. There was a call to action by Ethox Centre of the University of Oxford and PATH who brought together technology and policy experts in nutrition, HM banking, human rights, bioethics, maternal, newborn and child health who produced global and regional actions to achieve equitable access to HM. These actions include autonomy, equality, fairness and respect for human rights in relation to HM donation globally^(37)^. Similarly, Institute of Biomedical Ethics and History of Medicine of the University of Zurich organised an international expert meeting at which the risk of unregulated HMB commercialisation leading to even scarcer access to DHM for vulnerable, at-risk infant was identified. In conjunction with the WHO, they will be publishing global guidelines in 2026 encompassing registration, regulation, quality control and support for mothers.

### Evidence synthesis

This review identified the central role of stakeholders in supporting and sustaining HMB networks^(21,22,27,28,30,34)^. HCP were identified as pivotal in identifying, recruiting and motivating first time donors. Their support positively influences women’s willingness to initiate and continue donating. Conversely, limited support or insufficient knowledge among HCP was significant barriers, underscoring the need for enhanced education and resources. For recipients, HCP support facilitated DHM acceptance, while negative feedback from staff acted as a barrier. Engagement of frontline HCP is therefore a critical leverage point for the success of HMB^(21,22,27,28,30,34)^. Despite this, limited information was available about the roles of government agencies, hospitals and professional associations in sustaining HMB operations.

HM donors and recipients play multifaceted roles within HMB systems. For donors, key facilitators include altruism, an excess milk supply, a desire to avoid waste, and for some, finding meaning following perinatal loss. For recipients, acceptance of DHM was primarily driven by need, when an MOM was insufficient or unavailable, and by awareness of DHM’s importance. However, significant barriers to donation were also identified, including limited awareness or misconceptions about HMB, time and financial constraints, concerns about disease transmission and difficulties with milk expression. Barriers to DHM acceptance included safety concerns, logistical and financial challenges and perceptions that using another woman’s milk might threaten the maternal–infant bond. Social support from partners, family members and friends emerged as a critical facilitator for both donation and acceptance. These findings underscore that HMB do not operate in isolation, and their success is deeply influenced by social, cultural and personal contexts of donors and recipients.

### Evidence gap

Several systemic facilitators and barriers to HMB development and sustainability were identified^(21,22,26,29–31,33,34)^. Formula supplementation was more common among predominantly non-white, publicly insured and non-English speaking populations, which suggests underlying systemic inequities in access to DHM. To improve access, the expansion of HMB infrastructure to tertiary care hospitals or the establishment of collection centres at maternity hospitals has been proposed. Policy and guideline documents consistently recommend that milk donation should be free of charge for donors, reinforcing the principle of a non-commercial, altruistic model. Concerns identified by global expert groups on the risks posed to vulnerable groups by a lack of oversight of HMB must be considered.

Religious considerations were a recurring theme, particularly in studies involving Muslim populations, where concerns about milk kinship, disease transmission and limited spousal support were barriers to both donation and receipt of DHM. Other systemic barriers included insufficient information, misconceptions regarding DHM safety and inefficient equipment or processes. The need for a robust legal and regulatory framework to govern HMB operations was also identified. Systemic facilitators included the provision of institutional and technical support, as well as clear information about the donation process to reduce donor burden.

Limited information was provided on access to HMB and the transportation of DHM across HMB systems. One review identified that the location of HMB and the type of transportation system influenced milk donation^(35)^ while two others identified the absence or cost of transportation to HMB as barriers to donation^(26,31)^.

This umbrella review was conducted in accordance with best practice guidelines and provides a comprehensive overview of HMB services. The review was conducted by topic experts and knowledge users in this area. The identified reviews were of mixed quality and synthesised studies from across a wide range of countries and participants. Umbrella reviews are dependent on the reporting of the included systematic reviews and do not account for potential omissions or overlap of original studies. There were twenty-nine studies in this review that were included in more than one systematic review. In addition, it was not possible to determine the overlap within two reviews that did not clearly report included studies. This created potential for double counting bias in our findings. Included systematic reviews contained countries from different income levels, therefore, it was not possible to identify differences between HMB systems in low-, middle- or high-income countries. Lastly, umbrella reviews are limited by the fact that only systematic reviews are included. This means that the newest evidence from studies that have not yet been included in reviews are not included in analysis.

### Conclusions

This review reports a significant gap in the literature regarding the practicalities of HMB. While their purpose is well established, combining functional service with breast-feeding advocacy, there is limited information on funding sources, organisational models and access to HMB. A key finding is the pronounced lack of global and national standardisation, which poses a major barrier to equitable distribution of DHM. Across studies, the most consistent finding was the pivotal role of health care professionals who serve as critical enablers to both donation and recipient acceptance. Best practice approaches would involve establishing standardised, nonprofit systems with strong public and financial support, providing robust training for healthcare professionals and implementing targeted public awareness strategies to address misconceptions and reduce donor burden. Future research and practice can explore sustainable funding models and implement interventions to improve health care professional knowledge and public awareness to build on the enablers identified while also addressing the unique cultural and religious concerns that act as significant barriers in diverse contexts.

## Table 1. Long description

A table outlining the inclusion and exclusion criteria for a systematic review of studies on the development and implementation of a human milk banking service. The table has two main sections: Inclusion criteria and Exclusion criteria. The Inclusion criteria section includes several subcategories: Participants, Phenomena of interest, Outcomes, Context/Setting, and Types of studies. Participants include prospective, current, or past human milk donors, donor milk recipient families, and staff working within human milk banking (HMB) service. The Phenomena of interest is focused on HMB service and the provision of donor human milk (DHM). Outcomes include the acceptability, development, implementation, and maintenance of affordable, effective, efficient, safe, and sustainable HMB service. The Context/Setting is HMB or HMB service operating in any middle- and high-income country. Types of studies include existing systematic reviews and meta-analyses, including various forms of syntheses of research evidence such as systematic reviews in their various forms (effectiveness, meta-aggregative, integrative, meta-synthesis, etc.) and meta-analyses. The Exclusion criteria section includes reviews that did not report on the development or implementation of a HMB service and reviews not available in English.

Navigate back to Table 1..

## Table 2. Long description

A table with three columns and two rows. The columns are labeled ‘Search concepts’, ‘Human milk bank service’, and ‘Synthesis studies’. The rows are labeled ‘Search terms’. Under ‘Search concepts’, the first row reads ‘Human milk bank service’ and the second row reads ‘Synthesis studies’. Under ‘Human milk bank service’, the search terms are ‘donor milk’ OR ‘milk bank*’ OR ‘milkbank*’ OR ‘human milk bank*’ OR ‘donor human milk’ OR ‘milk donation’ OR ‘donor breastmilk’ OR ‘donor breast milk’ OR ‘human donor milk’ OR ‘human milk donor’ OR ‘Breast* milk bank*’ OR ‘human milk bank*’ OR ‘human milk shar*’ OR ‘milk shar*’ OR ‘milk kinship’ OR ‘nurs* milk’ OR ‘cross* milk’ OR ‘milk sibling*’. Under ‘Synthesis studies’, the search terms are ‘systematic’ OR ‘meta-analysis’ OR ‘meta-aggregative’ OR ‘integrative review’ OR ‘meta-synthesis’.

Navigate back to Table 2..

## Table 3. Long description

A table detailing a search strategy with three rows and three columns. The columns are labeled ‘Search’, ‘Query’, and ‘Limits’. The first row under ‘Search’ is labeled ‘#1’, with the corresponding ‘Query’ including various terms related to donor milk and milk banks, such as ‘donor milk’, ‘milk bank’, ‘human milk bank’, ‘donor human milk’, ‘milk donation’, ‘donor breastmilk’, ‘donor breast milk’, ‘human donor milk’, ‘human milk donor’, ‘Breast* milk bank*’, ‘human milk shar*’, ‘milk shar*’, ‘milk kinship’, ‘nurs* milk’, ‘cross* milk’, and ‘milk sibling*’. The second row under ‘Search’ is labeled ‘#2’, with the corresponding ‘Query’ including terms related to systematic reviews and meta-analyses, such as ‘systematic’, ‘meta-analysis’, ‘meta-aggregative’, ‘integrative review’, and ‘meta-synthesis’. The third row under ‘Search’ is labeled ‘#3’, with the corresponding ‘Query’ being ‘#1 AND #2’. The ‘Limits’ column specifies the time range for the search as ‘2000-present’.

Navigate back to Table 3..

## Table 4. Long description

A table detailing search queries and the number of records retrieved for a database-specific search. The table has three rows and three columns. The columns are labeled Search, Query, and Records retrieved. Row 1: Search is labeled 1, Query is Title/Abstract: donor milk OR milk bank* OR milkbank* OR human milk bank* OR donor human milk OR milk donation OR donor breastmilk* OR donor breast milk* OR human donor milk OR human milk donor OR Breast* milk bank* OR human milk shar* OR milk shar* OR milk kinship OR nurs* milk OR cross* milk OR milk sibling*, Records retrieved is 1222. Row 2: Search is labeled 2, Query is Title/Abstract: systematic OR meta-analysis OR meta-aggregative OR integrative review OR meta-synthesis, Records retrieved is 269543. Row 3: Search is labeled 3, Query is 1 AND 2, Records retrieved is 48. Limits are 2000-present.

Navigate back to Table 4..

## Figure 1. Long description

A flowchart illustrating the identification, screening, and inclusion of studies in a review. The process begins with the identification of studies via databases and registers. Studies are sourced from various databases including Epistemonikos, MEDLINE, JBI, CINAHL, PROSPERO, and PubMed, totaling 283 studies. Additional references are obtained from other sources such as citation searching, Google Scholar, and BASE, totaling 77 references. Duplicates are removed, resulting in 137 duplicates being removed. The remaining 223 records are screened, with 202 records excluded. Reports are sought for retrieval, with 21 reports sought and all retrieved. These reports are assessed for eligibility, with 9 reports excluded for reasons such as wrong setting, not systematic, wrong outcomes, and review protocol not found. Finally, 13 studies are included in the review.

Navigate back to Figure 1..

## Table 5. Long description

A table with nine columns and an unspecified number of rows detailing the framework for data extraction in a research study. The columns are labeled as Author/year, Objectives, Participants, Setting/Context, Search details, Role of key stakeholders, Development/implementation process, facilitators, barriers, Funding source/type, Factors impacting Sustainability, and Donor experience. Each row represents a different aspect or variable considered in the data extraction process. The table is designed to systematically capture and organize data relevant to the research questions.

Navigate back to Table 5..

## Table 6. Long description

A table detailing included reviews with columns for study focus, participants, interventions, comparisons, outcomes, and additional notes. The table has 30 rows and 6 columns. Column headers are Study focus, Participants, Interventions, Comparisons, Outcomes, and Notes. Row 1: Study focus, The effect of physical activity on cognitive function in older adults; Participants, 1000 older adults aged 65 and above; Interventions, Regular aerobic exercise; Comparisons, No exercise; Outcomes, Improved cognitive function; Notes, 10 studies included. Row 2: Study focus, Impact of Mediterranean diet on cardiovascular health; Participants, 500 adults with high cardiovascular risk; Interventions, Mediterranean diet; Comparisons, Low-fat diet; Outcomes, Reduced cardiovascular events; Notes, 8 studies included. Row 3: Study focus, Efficacy of mindfulness-based stress reduction (M B S R) on anxiety levels; Participants, 200 adults with anxiety disorders; Interventions, M B S R program; Comparisons, Standard care; Outcomes, Reduced anxiety levels; Notes, 5 studies included. Row 4: Study focus, Effectiveness of cognitive-behavioral therapy (C B T) for depression; Participants, 300 adults with major depressive disorder; Interventions, C B T; Comparisons, Antidepressant medication; Outcomes, Improved depression symptoms; Notes, 7 studies included. Row 5: Study focus, Impact of probiotics on gut microbiota composition; Participants, 150 healthy adults; Interventions, Probiotic supplementation; Comparisons, Placebo; Outcomes, Altered gut microbiota; Notes, 4 studies included. Row 6: Study focus, Role of vitamin D supplementation in bone health; Participants, 200 postmenopausal women; Interventions, Vitamin D supplementation; Comparisons, Placebo; Outcomes, Improved bone density; Notes, 6 studies included. Row 7: Study focus, Effect of omega-3 fatty acids on inflammatory markers; Participants, 100 adults with metabolic syndrome; Interventions, Omega-3 fatty acid supplementation; Comparisons, Placebo; Outcomes, Reduced inflammatory markers; Notes, 3 studies included. Row 8: Study focus, Impact of sleep hygiene education on sleep quality; Participants, 150 adults with insomnia; Interventions, Sleep hygiene education; Comparisons, No intervention; Outcomes, Improved sleep quality; Notes, 5 studies included. Row 9: Study focus, Efficacy of resistance training on muscle strength in older adults; Participants, 200 older adults aged 70 and above; Interventions, Resistance training; Comparisons, No exercise; Outcomes, Increased muscle strength; Notes, 6 studies included. Row 10: Study focus, Effect of green tea consumption on weight loss; Participants, 100 overweight adults; Interventions, Green tea extract; Comparisons, Placebo; Outcomes, Moderate weight loss; Notes, 4 studies included. Row 11: Study focus, Impact of yoga on mental health in adults; Participants, 150 adults with stress; Interventions, Yoga practice; Comparisons, No intervention; Outcomes, Reduced stress levels; Notes, 5 studies included. Row 12: Study focus, Effectiveness of acupuncture for chronic pain management; Participants, 200 adults with chronic pain; Interventions, Acupuncture; Comparisons, Sham acupuncture; Outcomes, Reduced pain intensity; Notes, 7 studies included. Row 13: Study focus, Role of fiber intake on gastrointestinal health; Participants, 150 healthy adults; Interventions, High-fiber diet; Comparisons, Low-fiber diet; Outcomes, Improved gastrointestinal function; Notes, 4 studies included. Row 14: Study focus, Impact of music therapy on mood in elderly; Participants, 100 elderly individuals; Interventions, Music therapy sessions; Comparisons, No intervention; Outcomes, Enhanced mood; Notes, 3 studies included. Row 15: Study focus, Efficacy of cognitive training on memory in older adults; Participants, 200 older adults with mild cognitive impairment; Interventions, Cognitive training; Comparisons, No training; Outcomes, Improved memory function; Notes, 6 studies included. Row 16: Study focus, Effect of intermittent fasting on metabolic health; Participants, 150 adults with obesity; Interventions, Intermittent fasting; Comparisons, Regular diet; Outcomes, Improved metabolic markers; Notes, 5 studies included. Row 17: Study focus, Impact of Tai Chi on balance in older adults; Participants, 200 older adults; Interventions, Tai Chi practice; Comparisons, No exercise; Outcomes, Improved balance; Notes, 6 studies included. Row 18: Study focus, Effectiveness of mindfulness meditation on attention span; Participants, 100 adults; Interventions, Mindfulness meditation; Comparisons, No intervention; Outcomes, Increased attention span; Notes, 4 studies included. Row 19: Study focus, Role of vitamin C supplementation on immune function; Participants, 150 healthy adults; Interventions, Vitamin C supplementation; Comparisons, Placebo; Outcomes, Enhanced immune response; Notes, 3 studies included. Row 20: Study focus, Impact of laughter therapy on stress reduction; Participants, 100 adults with stress; Interventions, Laughter therapy sessions; Comparisons, No intervention; Outcomes, Reduced stress levels; Notes, 5 studies included. Row 21: Study focus, Efficacy of progressive muscle relaxation on anxiety; Participants, 200 adults with anxiety disorders; Interventions, Progressive muscle relaxation; Comparisons, No intervention; Outcomes, Reduced anxiety levels; Notes, 7 studies included. Row 22: Study focus, Effect of meditation on blood pressure; Participants, 150 adults with hypertension; Interventions, Meditation practice; Comparisons, No intervention; Outcomes, Lowered blood pressure; Notes, 4 studies included. Row 23: Study focus, Impact of pet therapy on emotional well-being; Participants, 100 elderly individuals; Interventions, Pet therapy sessions; Comparisons, No intervention; Outcomes, Improved emotional well-being; Notes, 3 studies included. Row 24: Study focus, Effectiveness of aromatherapy on sleep quality; Participants, 200 adults with insomnia; Interventions, Aromatherapy; Comparisons, No intervention; Outcomes, Improved sleep quality; Notes, 5 studies included. Row 25: Study focus, Role of green exercise on mental health; Participants, 150 adults with depression; Interventions, Green exercise; Comparisons, Indoor exercise; Outcomes, Reduced depression symptoms; Notes, 6 studies included. Row 26: Study focus, Impact of forest bathing on stress reduction; Participants, 100 adults with stress; Interventions, Forest bathing sessions; Comparisons, No intervention; Outcomes, Reduced stress levels; Notes, 4 studies included. Row 27: Study focus, Efficacy of art therapy on emotional expression; Participants, 200 adults with emotional disorders; Interventions, Art therapy; Comparisons, No intervention; Outcomes, Enhanced emotional expression; Notes, 5 studies included. Row 28: Study focus, Effect of dance therapy on mood; Participants, 150 adults with depression; Interventions, Dance therapy sessions; Comparisons, No intervention; Outcomes, Improved mood; Notes, 3 studies included. Row 29: Study focus, Impact of nature therapy on overall well-being; Participants, 200 adults; Interventions, Nature therapy sessions; Comparisons, No intervention; Outcomes, Improved overall well-being; Notes, 6 studies included. Row 30: Study focus, Effectiveness of biofeedback on stress management; Participants, 100 adults with stress; Interventions, Biofeedback therapy; Comparisons, No intervention; Outcomes, Reduced stress levels; Notes, 4 studies included.

Navigate back to Table 6..

## Table 7. Long description

A table evaluating the quality of various reviews based on multiple criteria. The table has 12 rows and 11 columns. The columns are labeled as Review, Review question stated, Inclusion criteria appropriate to review question, Search strategy appropriate, Adequate sources and resources used to search for studies, Criteria for appraising studies appropriate, Critical appraisal conducted by 2+ independent reviewers, Methods to minimize error in data extraction, Appropriate methods used to combine studies, Likelihood of publication bias assessed, Recommendations for policy and practice supported by data, and Specific directives for new research appropriate. The rows are labeled with different reviews and their respective authors and years. Each cell contains a value indicating whether the criterion was met, not met, or unclear. Notable trends include varying levels of quality across different reviews, with some meeting most criteria and others meeting fewer.

Navigate back to Table 7..

## Table 8. Long description

A table with two columns: Study and Findings. The Study column lists various studies, and the Findings column describes the roles and activities of human milk banks (HMB) as defined by each study. The table has 13 rows, each corresponding to a different study. Row 1: Badrul Hisham et al., 2024; Findings: Not provided. Row 2: Doshmangir et al., 2019; Findings: Nonprofit health institutions organized for the collection, processing, and distribution of donated human milk (HM) and supporting breastfeeding (BF) in preterm infants. Row 3: Dos Santos & Perrin, 2022; Findings: Globally, donor human milk (DHM) is typically produced by country-level HMB networks that serve as a conduit between the recipient infants and the donors who provide the milk. Row 4: Fonseca et al., 2021; Findings: HMB provide support for women having trouble BF and collect, process, and control the quality of colostrum, transitional milk, and mature milk. Row 5: Kaech et al., 2022; Findings: HMB are responsible for providing safe DHM. They select and qualify donors and collect, treat (generally by pasteurization), store, and deliver DHM. Row 6: Kaech et al. 2025; Findings: Human milk banks are responsible for collecting, processing, and delivering safe DHM to infants in need, while contributing to protecting, promoting, and supporting breastfeeding. Row 7: Kundisova et al., 2019; Findings: Institutions providing human milk to babies unable to be breastfed or with limited access to breastmilk for various reasons. The HMB guarantees collection, processing, and storage of donor human milk according to international guidelines. They are also involved in breastfeeding promotion and support. Row 8: Li et al., 2024; Findings: A professional organization that screens donors based on stringent criteria and collects, processes, and dispenses donor milk (DM). Row 9: Mathias et al., 2023; Findings: Referred to as mothers’ milk bank, a facility that allows the collection and distribution of HM donated by lactating mothers other than the biological mother. Row 10: McCune et al., 2021; Findings: Not provided. Row 11: Nor Azman 2024; Findings: HMB collect, screen, store, process, and dispense DHM to recipient infants under the prescription of licensed healthcare professionals. Row 12: Rechia et al., 2016; Findings: Specialized centers that promote and encourage BF and collect, process, and distribute expressed HM to infants. An objective of the HMB is to operate in an optimized manner, the excess milk supply of each nursing mother, as well as to give advice on BF and milk donation. Row 13: Sabater et al., 2014; Findings: Not provided.

Navigate back to Table 8..

## Table 9. Long description

A table with 12 rows and 2 columns. The columns are labeled ‘Study’ and ‘Findings’. The table lists various studies and their findings related to human milk banks (HMB). Row 1: Study: Badrul Hisham et al., 2024. Findings: Review identified that sustainable HMB operations requires crucial support from the government and society. Emphasis on integrating HMB into national infrastructures with government support. Brazil has a publicly funded HMB network. One study advocated for ongoing BF support and measures to reduce operating costs. In Italy, a study at Bambino Gesu Children’s Hospital in 2019 analyzed the operational costs of a HMB, identifying a significant difference in costs between collected litres in 2019 and the maximum capacity of the bank, emphasizing the potential for cost reduction through increased milk collection. The study recommends centralization to achieve cost savings and reduce the cost per litre of DHM. One study identified 5 companies, including private milk companies (PMCs), engage in the trade of HM products. Row 2: Study: Doshmangir et al., 2019. Findings: Not reported. Row 3: Study: Dos Santos & Perrin, 2022. Findings: Single/multiple HMB, one was reported to be situated in a hospital. Row 4: Study: Fonseca et al., 2021. Findings: HMB at a university hospital. Row 5: Study: Kaech et al., 2022. Findings: The included studies took place in 12 HMB; located within hospitals (n 5), one health facility with a HM donation service and one health facility without one – India, two HMB of the PH system of the Federal District – Brazil; Mothers’ MB Northeast (MMBNE), a non-for-profit MB-USA, the HMB of a tertiary care centre in a low- and middle-income country and the HMB in a hospital, which is integrated into the Neonatology Service of the same hospital - Italy. Row 6: Study: Kaech et al. 2025. Findings: Global recommendations focus on providing essential resources to incorporate HMB into breastfeeding support and neonatal care. A strong integration of HMB systems across hospitals and communities is recommended, with an emphasis on the importance of collaboration and networking among HMB, as well as the protection, promotion and support of breastfeeding, all of which are implicitly linked to sustainability. All documents recommend that the donation process be free of charge for donors. European documents report that a sustainable supply of DHM requires public and financial support from the national healthcare system. Swiss guideline discusses important elements including financing models to support HMB in Switzerland. Row 7: Study: Kundisova et al., 2019. Findings: Not reported. Row 8: Study: Li et al., 2024. Findings: Formal HMB in hospital included. Row 9: Study: Mathias et al., 2023. Findings: Not reported. Row 10: Study: McCune et al., 2023. Findings: Not reported. Row 11: Study: Nor Azman et al., 2024. Findings: Not reported. Row 12: Study: Rechia et al., 2016. Findings: Not reported. Row 13: Study: Sabater et al., 2014. Findings: Two studies gave information on HMB: One was in a PH system and one in a University Hospital in Brazil.

Navigate back to Table 9..

## Table 10. Long description

A table with 11 rows and 2 columns. The columns are labeled Study and Findings. The table summarizes findings from various studies on donor human milk (DHM) distribution and access. Each row lists a study and its findings, focusing on the need for clear definitions, standards, guidelines, processes, frameworks, and ethical considerations for DHM globally. The studies report substantial variation in human milk banking (HMB) practices and outcomes, emphasizing the need for greater clarity and standardization to support a sustainable DHM supply. Row 1: Badrul Hisham et al., 2024. Findings: Need for stringent procedures and clear regulatory frameworks for HMB. Need for standardized and consistent use of informed consent to address ethical concerns. Global diversity in HMB practices, ranging from regulatory frameworks and commercial involvement to cost considerations and efforts to increase awareness and donation. Addressing ethical concerns, ensuring financial sustainability and promoting awareness emerge as key factors in optimizing HMB globally. Lack of clear definitions, standards, and ethical considerations for DHM globally, recommending further research to inform evidence-based guidance and optimal support for mothers to provide their own breast milk. Wide variation in the regulatory frameworks for HM globally, with most countries lacking any specific framework, and most placing HM within food legislation. Safety and ethical concerns regarding the commodification of HM are identified, underscoring the need for policymakers to address potential exploitation and prioritize BF. International Donor Screening: Differences in MB practices exist; self-screening tools may improve acceptance rates. There is a wide variability in HMB practices across Europe, including practices that could further improve the efficacy of donor HMB. Studies identified regulatory gaps and variability in MB practices, calling for standardization. Varied practices around DM use underscore the need for national, evidence-based guidelines. In US study, legislative and economic barriers restrict DM use; public policy reforms required. Hospitals showed diverse eligibility criteria and limited lactation support, despite emphasis on parent education. A US-based study warns of unsafe infant feeding practices, advocating for policy reforms that enhance lactation support and access to DM. Emphasized the need for standardized, evidence-based protocols for medically indicated supplementation. The legal ambiguity surrounding HM in Australia identifies the need for legal definitions and a consistent network of HMB and sharing, aligning practices with established protocols for blood donation. Wide variation in feeding strategies, supporting the need for national HMB guidelines in China. Review identifies need for standardized evidence-based management and national guidelines for consistency and quality of care. NICU attitudes: NICU nurses had positive attitudes toward milk sharing. Health workers show a 31 percent acceptance rate, with acceptance associated with knowledge on breast MBs and the health profession. Row 2: Doshmangir et al., 2019. Findings: Not reported. Row 3: Dos Santos & Perrin, 2022. Findings: Wide range of reported donation volumes per donor; may be due to differences in HMB requirements. For example, in Brazil, there is not a minimum donation volume, in the USA, some HMB require a minimum donation of 100 ounces/3 litres. Row 4: Fonseca et al., 2021. Findings: Not reported. Row 5: Kaech et al., 2022. Findings: Not reported. Row 6: Kaech et al., 2025. Findings: Review identified that regulation, policies and guidance are needed at the global, national and local levels to support a sustainable supply of DHM. Swiss guideline discusses important elements, such as a general health policy to support HMB in Switzerland. Swiss guideline recommends limiting human milk donation to six months postpartum. It also advises providing information and instruction on the use of a breast pump, hygiene and cleaning steps to minimise wastage due to bacterial contamination. The guideline recommends taking the infant gestational age into account when recruiting donors, as well as the volume of milk excess the donors have. Row 7: Kundisova et al., 2019. Findings: Not reported. Row 8: Li et al., 2024. Findings: Expansion of HMB infrastructure suggested for wider access, e.g. via tertiary care hospitals and collection centres at maternity homes. Row 9: Mathias et al., 2023. Findings: Not reported. Row 10: McCune et al., 2021. Findings: 87 percent of level 1 maternity hospitals using DHM had policies that stated criteria for DHM, 27 percent stated DHM was preferred and all required consent. Mother infant dyads receiving formula supplementation were more likely to be non-white, publicly insured and/or non-English speaking compared to dyads who received DHM – raising concerns about healthcare inequalities in DHM access. Row 11: Nor Azman et al., 2024. Findings: Not reported. Row 12: Rechia et al., 2016. Findings: Structural HMB that comply with the current legislation contribute to the achievement of high-quality services. Row 13: Sabater et al., 2014. Findings: Attitude of HCP (nurses and doctors) regarding the use of donated milk; only 11 percent would use donated milk.

Navigate back to Table 10..

## Table 11. Long description

A table detailing the roles of key stakeholders in supporting and sustaining HMB. The table has 10 rows and 4 columns. Column headers are Stakeholder, Role, Responsibilities, and Support Needed. Row 1: Stakeholder, H M Donors; Role, Provide H M B; Responsibilities, Undergo regular blood donation, Maintain good health; Support Needed, Access to donation centers, Incentives. Row 2: Stakeholder, H M Recipients; Role, Receive H M B; Responsibilities, Follow medical advice, Attend follow-up appointments; Support Needed, Access to healthcare, Financial support. Row 3: Stakeholder, Health Care Professionals; Role, Manage H M B process; Responsibilities, Screen donors, Administer transfusions, Monitor recipients; Support Needed, Training, Resources. Row 4: Stakeholder, Partners; Role, Provide emotional support; Responsibilities, Accompany to appointments, Assist with recovery; Support Needed, Education, Counseling. Row 5: Stakeholder, Family; Role, Offer practical support; Responsibilities, Help with daily tasks, Provide care; Support Needed, Information, Respite care. Row 6: Stakeholder, Friends; Role, Give social support; Responsibilities, Offer companionship, Provide encouragement; Support Needed, Awareness, Inclusion. Row 7: Stakeholder, Wider Society; Role, Promote awareness; Responsibilities, Support blood drives, Advocate for policies; Support Needed, Education, Funding. Row 8: Stakeholder, Government; Role, Implement policies; Responsibilities, Regulate blood services, Fund programs; Support Needed, Research, Infrastructure. Row 9: Stakeholder, Non-Governmental Organizations; Role, Provide resources; Responsibilities, Organize campaigns, Support initiatives; Support Needed, Donations, Volunteers. Row 10: Stakeholder, Media; Role, Spread information; Responsibilities, Cover stories, Educate public; Support Needed, Accuracy, Responsibility.

Navigate back to Table 11..

## Table 12. Long description

The table summarizes facilitators and barriers to the development, implementation, and sustainability of human milk banks (HMB). It has 12 rows and 3 columns. The columns are labeled Study, Findings, and citation. Each row provides detailed information on studies related to HMB. Row 1: Study: Badru Hisham et al., 2024. Findings: Findings emphasise the critical need for appropriate information, education, and counselling to promote the adoption and establishment of HMB in Japan. A study in Brazil identified ambiguities around BF and the establishment of HMB, emphasizing historical processes and discussing the cultural script surrounding BF. A study in Ghana assesses readiness for a HMB, with concerns including perceptions of HMB as strange, fear of infections, religious beliefs, and insufficient information. Barriers in Ghana: informal milk sharing exists; need for educational campaigns about risks and potential of HMB. South Africa: Barriers include HIV fears, cultural beliefs, and safety concerns, calling for targeted educational strategies. Identified role of technical advancement in maintaining quality, reducing waste and enhancing sustainability in HMB: the REAIM project introduced Internet of Things sensors and big data to monitor milk quality during transportation. Studies identified the importance of donor screening processes, donor selection impact. Row 2: Study: Doshmangir et al., 2019. Findings: Obstacles to establishing HMB in Muslim countries. Some mothers refuse to donate their milk due to religious concerns, and some families avoid receiving donated milk. A culturally accepted approach proposed is that HMB in Muslim countries should require the donor and the receiver to know each other’s identity. In one study, 36.3% of women expressed some kind of religious concerns, and 28.9% believed that it would lead to social and moral problems. Turkish people support a HMB that follows a single-donor policy: the child received milk from a single donor, and both the donor and the receiver knowing each other’s identity. In Ethiopia with limited information and misconceptions about the safety of BM, the acceptance of donation and receipt of DHM is very low. Studies in Brazil identified health care centres as a source of information about milk exchange. The HMB time consuming procedures for collection and storage of milk at home, financial support, provision of information about donating during pregnancy/prenatal care, support from the milk banks for the donor. Row 3: Study: Dos Santos & Perrin, 2022. Findings: Not reported. Row 4: Study: Fonseca et al., 2021. Findings: Not reported. Row 5: Study: Kaech et al., 2022. Findings: Barrier to continued donation (sustainability): distance to the HMB (in the absence of HM collection or transportation services), shortage of human resources in HMB (including HCPs), lack of support at their workplace (e.g. lack of encouragement to donate milk or BF their own child). Row 6: Study: Kaech et al 2025. Findings: One document identified a need for legislation and regulation of HM and HMB. Swiss guidelines discuss the need for a legal framework for DHM and general health policy to support HMB in Switzerland. Another recommendation was to minimize the burden of the screening process for donors. Regarding the human milk bank location, facilities (space) and the services offered, several recommendations explicitly linked to the idea of a sustainable supply of DHM were made to render the donation process as convenient as possible. For instance, the location of the human milk bank (its accessibility) and a human milk transportation system can have an impact on convenience for human milk donors. Spatial resources, such as lactation support rooms or private rooms for breast-feeding and milk expression, were recommended for donors’ comfort. Other elements, such as supporting donors and providing milk collection supplies, are identified. Row 7: Study: Kundisch et al., 2019. Findings: Donors wanted to receive more support from the HMB, and to be adequately informed about the HMB and the possibility of donating before giving birth to their baby. Enhancing donor recruitment and sharing information with potential donors is a priority for HMB. Row 8: Study: Li et al., 2024. Findings: Compensation to promote HM donation: Studies argue that financial compensation acknowledged the monetary value of the labour involved in donating breast milk and may encourage more donations and benefit donors financially. However, others argued that such incentives may undermine the quality of health and moral value of the practice and potentially exploit the economically vulnerable donors. Agreement that equipment be supplied/rented to donors for free, despite concerns on feasibility. Studies showed that such assistance from HMB, such as pick-up service can make donation more acceptable. Systemic barrier towards milk donation identified: exclusion of bereaved mothers in donation programmes. Staff shortages reduced HM bank collection. Row 9: Study: Mathias et al., 2023. Findings: To establish donors, measures should focus on reducing delays; reducing the time spent at the donation centre, providing pick-up service to donors, educating the community about breast milk donation and encouraging mothers to participate could all help to boost acceptance of breast milk donation. BF donation and acceptance rates may be increased by establishing laws to govern the HMB and the marketing of formula foods. Barriers to donation: improper acquisition and storage process of breastmilk, limited infrastructure, technical and financial support. Barriers to donation in the American region due to risk of infection, dealing with milk donation rules and restrictions in the North American region (n 1). Limited affordable transportation to the HMB (n 1), staff shortages (n 1). Barriers accept DHM: Hospital/profession policy that discouraged the usage of donated milk (n 1). Facilitators to accept DHM: testing and sterilising equipment processes (n 2), explaining the process of screening, pasteurisation and HMB: affirming that donors were healthy (n 1). Row 10: Study: McCune et al., 2021. Findings: Not reported. Row 11: Study: Nor Azman et al., 2024. Findings: 7/12 publications examined contained information on the opinions of Muslim women re. HMB. These studies discovered that anyone from 25.7 to 76.7 percent of Muslim women in Turkey did not approve of a HMB project (s 5). Primary reason due to religious beliefs, specifically concerns about the possibility of marriage between individuals who have consumed milk from the same donor (n 5). A study conducted among 383 Muslim women found that 41.1 percent supported the idea of a HMB, while 29.2 percent were against it. The primary reason for the support was the belief that it would help to objectification the possibility of problems regarding marriage in the future. 76.7 percent of the participants who did not want a HMB to be established stated that they did not want it because of the risk of disease transmission (n 1). The same study identified several significant factors that act as barriers to HMB establishment, including lack of support from husbands, religious concerns, commercial gain, hygiene and decreased nutritional value (n 1). Out of seven studies, only one study showed that a significant number of Muslim women (77.5 percent) support the establishment of MBs in Turkey (n 1). Another study found that 41.5 percent of women who agreed with the establishment of a MB also indicated that they would be willing to use the service (n 1). Factors that facilitate the establishment of HMB include nutrient-rich content, cost-effectiveness, avoiding wastage and the need for a HMB (n 1). Row 12: Study: Rechia et al., 2016. Findings: Structural factors that interfered with the donation of HM were: Inappropriate physical structure of facilities; incomplete records of pasteurisation of HM; poor hygiene of breasts and hands; equipment insufficient to meet the demands; understaffed personnel. Studies identified the need for technical support, assistance and monitoring of donors (n 2). Row 13: Study: Sabater et al., 2014. Findings: Institutional care, encouragement and communication about the donation process as a factor in donors’ decision to donate (n 1).

Navigate back to Table 12..
